# DIA-datasnooping and identifiability

**DOI:** 10.1007/s00190-018-1141-3

**Published:** 2018-04-09

**Authors:** S. Zaminpardaz, P. J. G. Teunissen

**Affiliations:** 1 Department of Spatial Sciences, GNSS Research Centre, Curtin University, Perth, Australia; 2 Department of Geoscience and Remote Sensing, Delft University of Technology, Delft, The Netherlands

**Keywords:** Detection, identification and adaptation (DIA), Datasnooping, Misclosure space partitioning, DIA estimator, Minimal detectable bias (MDB), Minimal identifiable bias (MIB), Probability of correct identification, Nonseparable hypotheses

## Abstract

In this contribution, we present and analyze datasnooping in the context of the DIA method. As the DIA method for the detection, identification and adaptation of mismodelling errors is concerned with estimation and testing, it is the combination of both that needs to be considered. This combination is rigorously captured by the *DIA estimator*. We discuss and analyze the DIA-datasnooping decision probabilities and the construction of the corresponding partitioning of misclosure space. We also investigate the circumstances under which two or more hypotheses are *nonseparable* in the identification step. By means of a theorem on the equivalence between the nonseparability of hypotheses and the inestimability of parameters, we demonstrate that one can forget about adapting the parameter vector for hypotheses that are nonseparable. However, as this concerns the *complete* vector and not necessarily functions of it, we also show that parameter functions may exist for which adaptation is still possible. It is shown how this adaptation looks like and how it changes the structure of the DIA estimator. To demonstrate the performance of the various elements of DIA-datasnooping, we apply the theory to some selected examples. We analyze how geometry changes in the measurement setup affect the testing procedure, by studying their partitioning of misclosure space, the decision probabilities and the minimal detectable and identifiable biases. The difference between these two minimal biases is highlighted by showing the difference between their corresponding contributing factors. We also show that if two alternative hypotheses, say ${\mathcal {H}}_{i}$ and ${\mathcal {H}}_{j}$, are nonseparable, the testing procedure may have different levels of sensitivity to ${\mathcal {H}}_{i}$-biases compared to the same ${\mathcal {H}}_{j}$-biases.

## Introduction

The DIA method for the detection, identification and adaptation of mismodelling errors combines estimation with testing. This combination of estimation and testing can be rigorously captured in the *DIA estimator* as introduced in (Teunissen 2017). The DIA method has already been widely employed in a variety of applications, such as the quality control of geodetic networks and the integrity monitoring of GNSS models, see, e.g., (DGCC 1982; Teunissen 1990; Salzmann 1995; Tiberius 1998; Perfetti 2006; Khodabandeh and Teunissen 2016; Zaminpardaz et al. 2015).

In this contribution, as an important example of multiple hypothesis testing, datasnooping (Baarda 1967, 1968; Teunissen 1985) is presented in the context of the DIA method. In doing so, we make use of the partitioning of misclosure space based on which we discuss the datasnooping decision probabilities and the construction of the corresponding DIA estimator. Through this partitioning, the distribution of the misclosure vector can be used to determine the correct detection (CD) and correct identification (CI) probabilities of each of the alternative hypotheses, as well as their corresponding minimal biases, the minimal detectable bias (MDB) and the minimal identifiable bias (MIB). We highlight their difference by showing the difference between their corresponding contributing factors. We also investigate the circumstances under which two or more hypotheses are *nonseparable* and discuss the relevant corrective actions including ‘remeasurement’, ‘adaptation’ or stating that the solution is ‘unavailable’. Of these, the adaptation step is the most involved and will be discussed in more detail.

This contribution is structured as follows. In Sect. 2, we briefly review the DIA method, describe the steps of DIA-datasnooping and define its corresponding DIA estimator. We hereby highlight the role played by the chosen partitioning of misclosure space. In Sect. 3, the decision probabilities of DIA-datasnooping are discussed, whereby between the following events are distinguished: correct acceptance (CA), false alarm (FA), correct/missed detection and correct/wrong identification. It is hereby highlighted that the MDB provides information about correct detection and *not* about correct identification. A high probability of correct detection does namely not necessarily imply a high probability of correct identification, unless one is dealing with the special case of having only one single alternative hypothesis.

As identification of hypotheses becomes problematic if the misclosure vector has the *same* distribution under *different* hypotheses, we study its consequences for the identification and adaptation steps in Sect. 4. We discuss the corrective actions one can choose from in terms of ‘remeasurement’, ‘adaptation’ or stating that the solution is ‘unavailable’. Of these, the adaptation step is the most involved. By means of a theorem on the equivalence between the nonseparability of hypotheses and the inestimability of parameters, we demonstrate that one can forget about adapting the *complete* vector of unknowns for hypotheses that are nonseparable. However, it is demonstrated that there may exist parameter functions for which adaptation is still possible. It is shown how this adaptation looks like and how it changes the structure of the DIA estimator.

To illustrate and explain the performance of the various elements of DIA-datasnooping, the theory is applied to selected examples in Sect. 5. The following three different cases are treated: height-difference observations of a leveling network, distance measurements of a horizontal geodetic network and pseudorange measurements between a single ground station and GPS satellites. We analyze how geometry changes in the measurement setup affect the testing procedure, including its partitioning of the misclosure space, and the corresponding CD probabilities (MDB) and CI probabilities (MIB). We also demonstrate that for a given bias-to-noise ratio and a false alarm probability, the ordering of the CD probabilities of the alternative hypotheses is not necessarily the same as that of their CI probabilities. It is also shown if two alternative hypotheses, say ${\mathcal {H}}_{i}$ and ${\mathcal {H}}_{j}$, are not distinguishable, that the testing procedure may have different levels of sensitivity to ${\mathcal {H}}_{i}$-biases compared to the same ${\mathcal {H}}_{j}$-biases. Finally, a summary and conclusions are given in Sect. 6.

## Detection, identification and adaptation (DIA)

### DIA in brief

We first formulate the null- and alternative hypotheses, denoted as ${\mathcal {H}}_{0}$ and ${\mathcal {H}}_{i}$, respectively. Let the observational model under the null hypothesis be given as

1 $$\begin{aligned} {\mathcal {H}}_{0}:\quad E(y)\;=\;Ax;\quad D(y)\;=\;Q_{yy} \end{aligned}$$

with *E*(.) the expectation operator, *D*(.) the dispersion operator, $y\in {\mathbb {R}}^{m}$ the normally distributed random vector of observables linked to the estimable unknown parameters $x\in {\mathbb {R}}^{n}$ through the design matrix $A\in {\mathbb {R}}^{m\times n}$ of rank $(A)=n$, and $Q_{yy}\in {\mathbb {R}}^{m\times m}$ the positive-definite variance–covariance matrix of *y*. The redundancy of ${\mathcal {H}}_{0}$ is $r = m -\mathrm{rank}(A) = m - n$.

The validity of the null hypothesis can be violated if the functional model and/or the stochastic model is misspecified. Here we assume that a misspecification is restricted to an underparametrization of the mean of *y*, which is the most common error that occurs when formulating the model. Thus, the alternative hypothesis ${\mathcal {H}}_{i}$ is formulated as

2 $$\begin{aligned} {\mathcal {H}}_{i}:\quad E(y)\;=\;Ax\,+\,C_{i}b_{i};\quad D(y)\;=\;Q_{yy} \end{aligned}$$

for some vector $C_{i}b_{i}\in {\mathbb {R}}^{m}/\{0\}$ such that $[A~~C_{i}]$ is a known matrix of full rank and rank $([A~~C_{i}])<m$. $C_{i}$ and $b_{i}$ will further be specified in detail in Sect. 2.2. The best linear unbiased estimator (BLUE) of *x* under ${\mathcal {H}}_{0}$ and ${\mathcal {H}}_{i}$ is, respectively, denoted by ${\hat{x}}_{0}$ and ${\hat{x}}_{i}$ and given as

3 $$\begin{aligned} {\hat{x}}_{0}\;=\;A^{+}\,y \quad ,\quad {\hat{x}}_{i}\;=\;{\bar{A}}^{+}_{i}\,y \end{aligned}$$

with $A^{+}=(A^{T}Q_{yy}^{-1}A)^{-1}A^{T}Q_{yy}^{-1}$ the BLUE-inverse of *A*, ${\bar{A}}_{i}^{+}=({\bar{A}}_{i}^{T}Q_{yy}^{-1}{\bar{A}}_{i})^{-1}{\bar{A}}_{i}^{T}Q_{yy}^{-1}$ the BLUE-inverse of ${\bar{A}}_{i}=P_{C_{i}}^{\perp }\,A$ and $P_{C_{i}}^{\perp }=I_{m}-C_{i}(C_{i}^{T}Q_{yy}^{-1}C_{i})^{-1}C_{i}^{T}Q_{yy}^{-1}$ being the orthogonal projector that projects, along the range space of $C_{i}$, onto the $Q_{yy}^{-1}$-orthogonal complement of the range space of $C_{i}$.

As one often will have to consider more than one single alternative hypothesis, the statistical model validation of ${\mathcal {H}}_{0}$ and *k* alternatives ${\mathcal {H}}_{i}$ ($i=1,\ldots ,k$) usually goes along the following three steps of detection, identification and adaptation (DIA) (Baarda 1968; Teunissen 1990).

1. *Detection* The validity of the null hypothesis is checked by virtue of an overall model test, without the need of having to consider a particular set of alternative hypotheses. If ${\mathcal {H}}_{0}$ is accepted, ${\hat{x}}_{0}$ is provided as the estimate of *x*.
2. *Identification* In case ${\mathcal {H}}_{0}$ is rejected, a search is carried out among the specified alternative hypotheses ${\mathcal {H}}_{i}$ ($i=1,\ldots ,k$) with the purpose of pinpointing the potential source of model error. In doing so, two decisions can be made. Either one of the alternative hypotheses, say ${\mathcal {H}}_{i}$, is confidently identified, or none can be identified as such, in which case an ‘undecided’ decision is made.
3. *Adaptation* In case ${\mathcal {H}}_{i}$ is confidently identified, it is chosen as the new null hypothesis. The ${\mathcal {H}}_{0}$-based inferences are then accordingly corrected and ${\hat{x}}_{i}$ is provided as the estimate of *x*. However, in case the ‘undecided’ decision is made, then the solution for *x* is declared ‘unavailable’.

All the information that is needed for the above three steps is contained in the *misclosure* vector $t\in {\mathbb {R}}^{r}$ given as

4 $$\begin{aligned} t\;=\;B^{T}y;\qquad Q_{tt}\;=\;B^{T}Q_{yy}B \end{aligned}$$

where the $m\times r$ matrix *B* is a basis matrix of the null space of $A^{T}$ (cf. 1), i.e., $A^{T}B=0$ and rank$(B)=r$, and $Q_{tt}$ is the variance matrix of *t*. Assuming that the observations are normally distributed as $y\overset{{\mathcal {H}}_{i}}{\sim }{\mathcal {N}}(Ax+C_{i}b_{i},Q_{yy})$ for $i=0,1,\ldots ,k$ and with $C_{0}b_{0}=0$, the misclosure vector is then distributed as

5 $$\begin{aligned} t\overset{{\mathcal {H}}_{i}}{\sim }{\mathcal {N}}(\mu _{t_{i}}=B^{T}C_{i}b_{i},Q_{tt}),\qquad \mathrm{for}~~~i=0,1,\ldots k \end{aligned}$$

As *t* is zero-mean under ${\mathcal {H}}_{0}$ and also independent of ${\hat{x}}_{0}$, it provides all the available information useful for validation of ${\mathcal {H}}_{0}$ (Teunissen 2017). Thus, an unambiguous testing procedure can be established through assigning the outcomes of *t* to the statistical hypotheses ${\mathcal {H}}_{i}$ for $i=0,1,\ldots ,k$.

### DIA-datasnooping

So far, no assumption was made about the structure of $C_{i}$ in (2). As the problem of screening observations for possible outliers is an important example of multiple hypothesis testing (see, e.g., Baarda 1968; Van Mierlo 1980; Hawkins 1980; Teunissen 1985; Parkinson and Axelrad 1988; Sturza 1988; Van der Marel and Kosters 1990; Su et al. 2014), we will restrict our attention to this important case. We further assume that only one observation at a time is affected by an outlier. Thus, in (2), $b_{i}$ is the scalar outlier and $C_{i}$ takes the form of a canonical unit vector $c_{i}\in {\mathbb {R}}^{m}$ having 1 as its *i*th entry and zeros elsewhere. This leads to having as many alternative hypotheses as the observations, i.e., $k=m$. This procedure of screening each individual observation for the presence of an outlier is known as *datasnooping* (Baarda 1968; Kok 1984). The corresponding DIA steps are specified as follows:

1. *Detection* Accept ${\mathcal {H}}_{0}$ if $t\in {\mathcal {P}}_{0}$ with
  6 $$\begin{aligned} {\mathcal {P}}_{0}= & {} \left\{ t\in {\mathbb {R}}^{r}|~\Vert t\Vert ^{2}_{Q_{tt}}\le k_{\alpha }\right\} \end{aligned}$$
  in which $\Vert \cdot \Vert ^{2}_{Q_{tt}}=(\cdot )^{T}Q_{tt}^{-1}(\cdot )$ and $k_{\alpha }$ is the user-chosen $\alpha$-percentage of the central Chi-square distribution with *r* degrees of freedom. If ${\mathcal {H}}_{0}$ is accepted, then ${\hat{x}}_{0}$ is provided as the estimate of *x*. Otherwise, go to step 2.
2. *Identification* Form Baarda’s test statistic as (Baarda 1967; Teunissen 2000)
  7 $$\begin{aligned} w_{i} \;=\; \dfrac{c^{T}_{t_{i}}Q_{tt}^{-1}t}{\sqrt{c^{T}_{t_{i}}Q_{tt}^{-1}c_{t_{i}}}},\quad i=1,\ldots ,k \end{aligned}$$
  in which $c_{t_{i}}=B^{T}c_{i}$. Since $c_{i}$ is a canonical unit vector, $c_{t_{i}}$ is then the *i*th column of matrix $B^T$. Select ${\mathcal {H}}_{i\ne 0}$ if $t\in {\mathcal {P}}_{i\ne 0}$ with
  8 $$\begin{aligned} {\mathcal {P}}_{i\ne 0}= & {} \left\{ t\in {\mathbb {R}}^{r}/{\mathcal {P}}_{0}|~|w_{i}|=\underset{j\in \{1,\ldots ,k\}}{\max }\;|w_{j}|\right\} \end{aligned}$$
3. *Adaptation* If ${\mathcal {H}}_{i}$ is selected, then ${\hat{x}}_{i}$ is provided as the estimate of *x*.

Note, since $t=B^{T}{\hat{e}}_{0}$, with ${\hat{e}}_{0}=y-A{\hat{x}}_{0}$, that the above procedure can be formulated by means of the least-squares residual vector ${\hat{e}}_{0}$ as well, thus providing a perhaps more recognizable form of the testing procedure (Teunissen 2000). Also note that we assume the variance–covariance matrix $Q_{yy}$ to be known. Variance-component estimation (Teunissen and Amiri-Simkooei 2008) with further modification of the partitioning of misclosure space would need to be included in case of unknown variance components. In the simplest case of a single unknown variance of unit weight, the datasnooping partitioning gets determined by only the $w_j$ statistics, which then will have a studentized distribution instead of a standard normal one (Koch 1999; Teunissen 2000).

Finally note that the vector of misclosures *t* is not uniquely defined. This, however, does not affect the testing outcome as both the detector $\Vert t\Vert ^{2}_{Q_{tt}}$ and Baarda’s test statistic $w_{i}$ remain invariant for any one-to-one transformation of the misclosure vector. Therefore, instead of *t*, one can for instance also work with

9 $$\begin{aligned} {\bar{t}}\;=\;Q_{tt}^{-\frac{1}{2}}\,t \end{aligned}$$

which, given (5), is distributed as ${\bar{t}}\overset{{\mathcal {H}}_{i}}{\sim }{\mathcal {N}}(\mu _{{\bar{t}}_{i}}=Q_{tt}^{-\frac{1}{2}}\mu _{t_{i}},I_{r})$. The advantage of using ${\bar{t}}$ over *t* lies in the ease of visualizing certain effects due to the identity-variance matrix of ${\bar{t}}$. We will make use of this in Sect. 5. The partitioning corresponding with ${\bar{t}}$ is then characterized through

10 $$\begin{aligned} \overline{{\mathcal {P}}}_{0}= & {} \left\{ {\bar{t}}\in {\mathbb {R}}^{r}|~\Vert {\bar{t}}\Vert ^{2}\le k_{\alpha }\right\} \end{aligned}$$

11 $$\begin{aligned} \overline{{\mathcal {P}}}_{i\ne 0}= & {} \left\{ {\bar{t}}\in {\mathbb {R}}^{r}/\overline{{\mathcal {P}}}_{0}|~|{\bar{c}}^{T}_{i}{\bar{t}}|=\underset{j\in \{1,\ldots ,k\}}{\max }\;|{\bar{c}}^{T}_{j}{\bar{t}}|\right\} \end{aligned}$$

with ${\bar{c}}_{i}=Q_{tt}^{-\frac{1}{2}}c_{t_{i}}/\Vert c_{t_{i}}\Vert _{Q_{tt}}$ being a unit vector and $\Vert \cdot \Vert ^{2}=(\cdot )^{T}(\cdot )$. As such, $\overline{{\mathcal {P}}}_{0}$ contains ${\bar{t}}$’s inside and on a zero-centered sphere with the radius of $\sqrt{k}_{\alpha }$ whereas $\overline{{\mathcal {P}}}_{i\ne 0}$ includes all ${\bar{t}}$’s outside the mentioned sphere which, among ${\bar{c}}_{j}$ for $j=1,\ldots , k$, make the smallest angle with ${\bar{c}}_{i}$. The border between $\overline{{\mathcal {P}}}_{i\ne 0}$ and $\overline{{\mathcal {P}}}_{j\ne 0}$ is then the locus of the vectors ${\bar{t}}\in {\mathbb {R}}^{r}/\overline{{\mathcal {P}}}_{0}$ which make the same angle with ${\bar{c}}_{i}$ and ${\bar{c}}_{j}$. Therefore, the partitioning of ${\mathbb {R}}^{r}$ is driven by $k_{\alpha }$ and the relative orientation of ${\bar{c}}_{j}$ for $j=1,\ldots , k$ with respect to each other.

### DIA estimator

As the above three steps show, DIA-datasnooping combines estimation with testing. By using a canonical model formulation and a partitioning of misclosure space, a unifying framework to rigorously capture the probabilistic properties of this combination was presented in Teunissen (2017). It was there also shown how the combined estimation-testing scheme could be captured in one single DIA estimator. The DIA estimator is a function of $${\hat{x}}_{j}$$$$(j=0, 1, \ldots , k)$$ and the misclosure vector *t*, and it is given as

12 $$\begin{aligned} {\bar{x}}\;=\;\sum \limits _{j=0}^{k}\;{\hat{x}}_{j}\,{p}_{j}(t) \end{aligned}$$

with $p_{j}(t)$ being the indicator function of region ${\mathcal {P}}_{j}$, i.e., $p_{j}(t)=1$ for $t\in {\mathcal {P}}_{j}$ and $p_{j}(t)=0$ elsewhere. As ${\bar{x}}$ is linear in ${\hat{x}}_{j}$, the DIA estimator of $\theta =F^{T}x$ with $F\in {\mathbb {R}}^{n\times p}$ is given as

13 $$\begin{aligned} {\bar{\theta }}\;=\;\sum \limits _{j=0}^{k}\;{\hat{\theta }}_{j}\,{p}_{j}(t) \end{aligned}$$

with $${\hat{\theta }}_{j}=F^{T}{\hat{x}}_{j}$$. For a general probabilistic evaluation of the DIA estimator, we refer to Teunissen (2017), but see also Teunissen et al. (2017). Here we note, however, that expressions (12) and (13) are only valid under the assumption that the set of regions $${\mathcal {P}}_{i}$$$$(i= 0, 1, \ldots , k)$$ forms a *partitioning* of misclosure space, i.e., $$\cup _{i=0}^{k}{\mathcal {P}}_{i}={\mathbb {R}}^{r}$$ and $${\mathcal {P}}_{i}\cap {\mathcal {P}}_{j}=\emptyset $$ for any $$i\ne j$$. Note the second condition is considered for the interior points of the distinct regions $${\mathcal {P}}_{i}$$. The regions $${\mathcal {P}}_{i}$$ are allowed to have common boundaries since we assume the probability of *t* lying on one of the boundaries to be zero. That the set of regions $${\mathcal {P}}_{i}$$$$(i= 0, 1, \ldots , k)$$ forms a *partitioning* of misclosure space requires that the canonical unit vectors of the individual hypotheses satisfy certain conditions.

#### Lemma 1

(Datasnooping partitioning) The $m+1$ regions ${\mathcal {P}}_{i}$ of (6) and (8) form a partitioning of misclosure space iff $c_{t_{i}}\nparallel c_{t_{j}}$ for any $i\ne j$.

#### Proof

See Appendix.$$\square $$

It will be clear that the conditions of the above Lemma may not always be fulfilled. The question is then which strategy to follow to deal with such a situation. Should one decide for ‘undecidedness’ if $c_{t_{i}} \parallel c_{t_{j}}$ for some $i \ne j$, or should one re-measure all such involved observables, or would it still be possible to perform an adaptation? An answer to these questions is provided in Sect. 4, where we consider the more general case and not restrict $C_{i}$ to be the canonical unit vector $c_{i}$. First, however, we discuss the testing probabilities that are involved in the detection and identification step.

## Detection versus identification

### The probabilities

As shown by (6), (7) and (8), the decisions of the testing procedure are driven by the outcome of the misclosure vector *t*. The probabilities of their occurrence depend on which of the hypotheses is true. If ${\mathcal {H}}_{i}$ is true, then the decision is correct if $t\in {\mathcal {P}}_{i}$, and wrong if $t\in {\mathcal {P}}_{j\ne i}$. We therefore discriminate between the following events

14 $$\begin{aligned} \begin{array}{lllll} \mathrm{CA}&{}=&{}(t\in {\mathcal {P}}_{0}|{\mathcal {H}}_{0})&{}=&{}\mathrm{correct~ acceptance}\\ \mathrm{FA}&{}=&{}(t\notin {\mathcal {P}}_{0}|{\mathcal {H}}_{0})&{}=&{}\mathrm{false~ alarm}\\ \mathrm{MD}_{i}&{}=&{}(t\in {\mathcal {P}}_{0}|{\mathcal {H}}_{i})&{}=&{}\mathrm{missed~ detection}\\ \mathrm{CD}_{i}&{}=&{}(t\notin {\mathcal {P}}_{0}|{\mathcal {H}}_{i})&{}=&{}\mathrm{correct~ detection}\\ \mathrm{WI}_{i}&{}=&{}(t\in \cup _{j\ne 0,i}^{k}{\mathcal {P}}_{j}|{\mathcal {H}}_{i})&{}=&{}\mathrm{wrong~ identification}\\ \mathrm{CI}_{i}&{}=&{}(t\in {\mathcal {P}}_{i}|{\mathcal {H}}_{i})&{}=&{}\mathrm{correct~ identification}\\ \end{array} \end{aligned}$$

With $*=\{\mathrm{CA,FA,MD}_{i},\mathrm{CD}_{i},\mathrm{WI}_{i},\mathrm{CI}_{i}\}$, we denote the probability of $*$ by $\mathrm{P}_{*}$ satisfying

15 $$\begin{aligned}&\mathrm{P}_{\mathrm{CA}}+\mathrm{P}_{\mathrm{FA}}=1 \nonumber \\&\mathrm{P}_{\mathrm{MD}_{i}}+\mathrm{P}_{\mathrm{CD}_{i}}=1 \nonumber \\&\mathrm{P}_{\mathrm{WI}_{i}}+\mathrm{P}_{\mathrm{CI}_{i}}=\mathrm{P}_{\mathrm{CD}_{i}} \end{aligned}$$

Computation of $\mathrm{P}_{*}$ requires information about the misclosures probability density function (PDF) which is given in (5). Here, it is important to note the difference between the CD and CI probabilities, i.e., $\mathrm{P}_{\mathrm{CD}_{i}}\ge \mathrm{P}_{\mathrm{CI}_{i}}$. They would be the same if there is only one alternative hypothesis, say ${\mathcal {H}}_{i}$, since then ${\mathcal {P}}_{i}={\mathbb {R}}^{r}/{\mathcal {P}}_{0}$. Analogous to the CD and CI probabilities, we have the concepts of the minimal detectable bias (MDB) (Baarda 1968) and the minimal identifiable bias (MIB) (Teunissen 2017). In the following, the difference between the MDB ($\mathrm{P}_{\mathrm{CD}_{i}}$) and the MIB ($\mathrm{P}_{\mathrm{CI}_{i}}$) is highlighted by showing the difference between their corresponding contributing factors.

### Minimal detectable bias (MDB)

The MDB of the alternative hypothesis ${\mathcal {H}}_{i}$ is defined as the smallest value of $|b_{i}|$ that can be detected given a certain CD probability. Therefore, the MDB is an indicator of the sensitivity of the *detection* step. Under ${\mathcal {H}}_{i\ne 0}$ with the definition of ${\mathcal {P}}_{0}$ in (6), the probability of correct detection reads

16 $$\begin{aligned} \mathrm{P}_{\mathrm{CD}_{i}}= & {} \mathrm{P}(t\notin {\mathcal {P}}_{0}|{\mathcal {H}}_{i}) =\mathrm{P}(\Vert t\Vert ^{2}_{Q_{tt}}>k_{\alpha }|{\mathcal {H}}_{i}) \end{aligned}$$

The MDB of ${\mathcal {H}}_{i}$ can then be computed by inverting the above equation for a certain CD probability. With (5), we have $\Vert t\Vert ^{2}_{Q_{tt}}\overset{{\mathcal {H}}_{i}}{\sim }\chi ^{2}(r,\lambda _{i}^{2})$ with $\lambda _{i}^{2}=\Vert \mu _{t_{i}}\Vert ^{2}_{Q_{tt}}$. For certain $\mathrm{P}_{\mathrm{FA}}=\alpha$, $\mathrm{P}_{\mathrm{CD}_{i}}=\gamma _{\mathrm{CD}}$ and *r*, one can compute $\lambda _{i}^{2}=\lambda ^{2}(\alpha ,\gamma _{\mathrm{CD}},r)$ from the Chi-square distribution, and then the MDB is (Baarda 1968; Teunissen 2000)

17 $$\begin{aligned} |b_{i,\mathrm{MDB}}|\;=\;\dfrac{\lambda (\alpha ,\gamma _{\mathrm{CD}},r)}{\Vert c_{t_{i}}\Vert _{Q_{tt}}} \end{aligned}$$

which shows that for a given set of $\{\alpha ,\gamma _{\mathrm{CD}},r\}$, the MDB depends on $\Vert c_{t_{i}}\Vert _{Q_{tt}}$. One can compare the MDBs of different alternative hypotheses for a given set of $\{\alpha ,\gamma _{\mathrm{CD}},r\}$, which provides information on how sensitive is the rejection of ${\mathcal {H}}_{0}$ for the biases the size of $|b_{i,\mathrm{MDB}}|^{,}$s. The smaller the MDB $|b_{i,\mathrm{MDB}}|$ is, the more sensitive is the rejection of ${\mathcal {H}}_{0}$.

### Minimal identifiable bias (MIB)

It is important to realize that the MDB provides information about correct detection and *not* correct identification. A high probability of correct detection does therefore not necessarily imply a high probability of correct identification (cf. 15), unless we have the special case of only a single alternative hypothesis. In case of multiple hypotheses, one can define the MIB of the alternative hypothesis ${\mathcal {H}}_{i}$ as the smallest value of $|b_{i}|$ that can be identified given a certain CI probability. It is an indicator of the sensitivity of the *identification* step. The MIB, denoted by $|b_{i,\mathrm{MIB}}|$, can be computed through inverting

18 $$\begin{aligned} \mathrm{P}_{\mathrm{CI}_{i}}= & {} \mathrm{P}(t\in {\mathcal {P}}_{i}|{\mathcal {H}}_{i})= \int _{{\mathcal {P}}_{i}}\;f_{t}(\tau |{\mathcal {H}}_{i})\;\mathrm{d}\tau \end{aligned}$$

for a given CI probability. The above probability is an *r*-fold integral over the complex region ${\mathcal {P}}_{i}$. Thus, the inversion of (18) is not as trivial as that of (16). The MIB needs then to be computed through numerical simulations, see, e.g., Teunissen (2017), pp. 73 and Robert and Casella (2013). From $\mathrm{P}_{\mathrm{CD}_{i}}\ge \mathrm{P}_{\mathrm{CI}_{i}}$, one can infer that $|b_{i,\mathrm{MDB}}|\le |b_{i,\mathrm{MIB}}|$ given $\mathrm{P}_{\mathrm{CI}_{i}}=\gamma _{\mathrm{CD}}$. The identification of mismodeling errors is thus more difficult than their detection (Imparato et al. 2018).

Although computation of (18) is not trivial, we can still assess the behavior of CI probability in relation to the contributing factors. To simplify such assessment, we make use of ${\bar{t}}$ instead of *t* and present the CI probability as

19 $$\begin{aligned} \mathrm{P}_{\mathrm{CI}_{i}}= & {} \mathrm{P}({\bar{t}}\in \overline{{\mathcal {P}}}_{i}|{\mathcal {H}}_{i})= \int _{\overline{{\mathcal {P}}}_{i}}\;f_{{\bar{t}}}(\tau |{\mathcal {H}}_{i})\;\mathrm{d}\tau \end{aligned}$$

With the definition of $\overline{{\mathcal {P}}}_{i}$ in (11) and $E({\bar{t}}|{\mathcal {H}}_{i})=(b_{i}\Vert c_{t_{i}}\Vert _{Q_{tt}}){\bar{c}}_{i}$, the CI probability, for a given value of $b_{i}$, is dependent on the following three factors

- $\overline{{\mathcal {P}}}_{i}$: As the integrand function in (19) is positive for all ${\tau }\in {\mathbb {R}}^{r}$, then the integral value will increase as $\overline{{\mathcal {P}}}_{i}$ expands.
- The orientation of ${\bar{c}}_{i}$ w.r.t. the borders of $\overline{{\mathcal {P}}}_{i}$: The unit vector ${\bar{c}}_{i}$, lying within the borders of $\overline{{\mathcal {P}}}_{i}$, determines the direction of $E({\bar{t}}|{\mathcal {H}}_{i})$ about which the PDF $f_{{\bar{t}}}({\tau }|{\mathcal {H}}_{i})$ is symmetric. The following lemma elaborates the role of the orientation of ${\bar{c}}_{i}$ in CI probability for $r=2$. For this case, the regions $\overline{{\mathcal {P}}}_{i}$ in (11) are defined in ${\mathbb {R}}^{2}$. Each region has then three borders of which one is curved (with $\overline{{\mathcal {P}}}_{0}$) and two are straight lines on either sides of ${\bar{c}}_{i}$.

#### Lemma 2

($\mathrm{P}_{\mathrm{CI}_{i}}$ as function of the orientation of ${\bar{c}}_{i}$) Let $\beta _{i}$ be the angle between the two straight borders of $\overline{{\mathcal {P}}}_{i}$ and let $\beta _{i,1}$ be the angle between ${\bar{c}}_{i}$ and the closest straight border on its right side (see Fig. 2). For a given $\beta _{i}$, $k_{\alpha }$ and $f_{{\bar{t}}}(\tau |{\mathcal {H}}_{i})$, the CI probability depends on $\beta _{i,1}$. We then have

20 $$\begin{aligned} \dfrac{1}{2}\beta _{i}\;=\;\underset{\beta _{i,1}}{\mathrm{argmax}}\;\mathrm{P}_{\mathrm{CI}_{i}}(\beta _{i,1}) \end{aligned}$$

#### Proof

See the Appendix. $\square$

Therefore, for $r=2$, for a given $\beta _{i}$, $k_{\alpha }$ and $f_{{\bar{t}}}(\tau |{\mathcal {H}}_{i})$, the CI probability reaches its maximum if ${\bar{c}}_{i}$ is parallel to the bisector line of the angle between the two straight borders of $\overline{{\mathcal {P}}}_{i}$.

- $\Vert c_{t_{i}}\Vert _{Q_{tt}}$: The scalar $\Vert c_{t_{i}}\Vert _{Q_{tt}}$ determines the magnitude of $E({\bar{t}}|{\mathcal {H}}_{i})$. Therefore, the larger the value of $\Vert c_{t_{i}}\Vert _{Q_{tt}}$, the further the center of $f_{{\bar{t}}}({\tau }|{\mathcal {H}}_{i})$ gets from the origin along ${\bar{c}}_{i}$, and the larger the probability mass of $f_{{\bar{t}}}({\tau }|{\mathcal {H}}_{i})$ inside $\overline{{\mathcal {P}}}_{i}$ will become.

We will use this insight in the contributing factors of the CI probability to explain some of the phenomena that we come across in our numerical analysis in Sect. 5.

## Nonseparable hypotheses

### Identifying nonseparable hypotheses

As any testing procedure is driven by the misclosure vector, identification of hypotheses becomes problematic if the misclosure vector has the *same* distribution under *different* hypotheses. According to (5) this happens when for two different hypotheses, say $${\mathcal {H}}_{i}$$ and $${\mathcal {H}}_{j}$$$$(i\ne j)$$,

21 $$\begin{aligned} B^{T}\,C_{i}\;=\;B^{T}\,C_{j}\,X_{i,j}\quad \mathrm{for~some~invertible~}~ X_{i,j}\in {\mathbb {R}}^{q\times q} \end{aligned}$$

In such a case, the misclosure vector *t* remains insensitive for the differences between ${\mathcal {H}}_{i}$ and ${\mathcal {H}}_{j}$, as a consequence of which we have ${\mathcal {P}}_{i}={\mathcal {P}}_{j}$. One can then not distinguish between the two hypotheses ${\mathcal {H}}_{i}$ and ${\mathcal {H}}_{j}$ in the identification step. If this is the case and $t \in {\mathcal {P}}_{i}={\mathcal {P}}_{j}$, one may consider the following:

1. *Remeasurement* If in case of datasnooping, ${\mathcal {H}}_{i}$ and ${\mathcal {H}}_{j}$ are singled out in the identification step, then it is one of the two observables, $y_{i}=c_{i}^{T}y$ or $y_{j}=c_{j}^{T}y$, that is suspected to contain a blunder or outlier. To remedy the situation, one may then decide to replace *both*$y_{i}$ and $y_{j}$ by their remeasured values.
2. *Adaptation* If remeasurement is not an option, one might think that adaptation of ${\hat{x}}_{0}$ would be an option by extending the design matrix to $[A~C_{i}~C_{j}]$, so as to cover both the hypotheses ${\mathcal {H}}_{i}$ and ${\mathcal {H}}_{j}$. But, as the theorem below shows, this is unfortunately not possible as *x* will then become inestimable. Also note, despite the nonseparability of the two hypotheses, that adaptation on either $[A~C_{i}]$ or $[A~C_{j}]$ should not be pursued. Such adaptation will still produce a biased result if done for the wrong hypothesis.
3. *Unavailability* Without remeasurement or adaptation, the remaining option is to declare a solution for *x* to be unavailable.

In the following theorem, we show an equivalence between the nonseparability of hypotheses and the inestimability of parameters.

#### Theorem 1

(Nonseparable hypotheses and inestimable parameters) Let [*A*  *B*] be an invertible matrix, with *A* of order $m\times n$ and *B* of order $m\times (m-n)$ satisfying $B^{T}A=0$. Furthermore, for any $i\ne j$ and $i,j=1,\ldots ,l$, let $C_{i}$ be full-rank matrices of order $m\times q$ with $m-n> q$ such that rank ($[C_{i}~~C_{j}]$)$> q$ and rank $([A~~C_{i}])=n+q$. Then for any $i\ne j$ and $i,j=1,\ldots ,l$, for some invertible matrix $X_{i,j}\in {\mathbb {R}}^{q\times q}$

22 $$\begin{aligned} B^{T}\,C_{i}\;=\;B^{T}\,C_{j}\,X_{i,j} \end{aligned}$$

iff

23 $$\begin{aligned} \exists ~ X\in {\mathbb {R}}^{n\times q}/\{0\}:~ [A~~C_{i}~~C_{j}]\,\left[ \begin{array}{c}X\\ ,I_{q}\\ X_{i,j}\end{array}\right] \;=\;0 \end{aligned}$$

implying that the extended design matrix $[A~~C_{i}~~C_{j}]$ is rank-deficient.

#### Proof

See the Appendix. $\square$

The above theorem conveys that if the alternative hypotheses ${\mathcal {H}}_{i}$ with $i=1,\ldots ,l$ are not distinguishable, then extending the design matrix *A* by *any* two or more matrices $C_{i}$ with $i=1,\ldots ,l$ will result in a rank-deficient design matrix and therefore make unbiased estimability of the parameter vector *x* impossible. The conclusion reads therefore that if remeasurement is not an option and *x* is the parameter vector for which a solution is sought, the issue of nonseparable hypotheses should already be tackled at the designing phase of the measurement experiment.

### Adaptation for estimable functions

The above theorem has shown that one can forget about adapting ${\hat{x}}_{0}$ for hypotheses that are nonseparable. This concerns, however, the *complete* vector *x* and not necessarily functions of *x*. It could still be possible that some relevant components of *x* or some relevant functions of *x* remain estimable, despite the rank-deficiency of the extended design matrix. The following theorem specifies which parameters remain estimable after the mentioned extension of the design matrix as well as presents the corresponding adaptation step for these estimable parameters.

#### Theorem 2

(Adaptation for nonseparable hypotheses) (i) Estimability: Let $C_{i}$, with $i=1,\ldots ,l$, be full-rank matrices of order $m\times q$ with $m-n> q$ satisfying (22) and (23). Also, let $C\in {\mathbb {R}}^{m\times l_{1}q}$ be a matrix formed by putting $l_{1}$ matrices $C_{i}$ column-wise next to each other. Then $\theta =F^{T}x$, with $F\in {\mathbb {R}}^{n\times p}$, is unbiased estimable under the extended model

24 $$\begin{aligned} E(y)\;=\;[A~~C]\left[ \begin{array}{l}x\\ b\end{array}\right] ;\quad D(y)\;=\;Q_{yy} \end{aligned}$$

iff

25 $$\begin{aligned} F^{T}V\;=\;0 \end{aligned}$$

in which *V* is a basis matrix of the null space of $C^{\perp ^{T}}A$, i.e., $C^{\perp ^{T}}A\,V=0$, and $C^{\perp }$ is a basis matrix of the orthogonal complement of the range space of *C*.

(ii) Adaptation: The BLUE of $\theta = F^{T}x$ under (24) and its variance matrix, denoted as ${\hat{\theta }}$ and $Q_{{\hat{\theta }}{\hat{\theta }}}$, respectively, can be written in adapted form as

26 $$\begin{aligned} {\hat{\theta }}= & {} {\hat{\theta }}_{0}\;+\;M\,y \nonumber \\ Q_{{\hat{\theta }}{\hat{\theta }}}= & {} Q_{{\hat{\theta }}_{0}{\hat{\theta }}_{0}}\;+\;M\,Q_{yy}\,M^{T} \end{aligned}$$

with ${\hat{\theta }}_{0}=F^{T}{\hat{x}}_{0}$, $Q_{{\hat{\theta }}_{0}{\hat{\theta }}_{0}}= F^{T}Q_{{\hat{x}}_{0}{\hat{x}}_{0}}F$ and where $M=F^{T}{\bar{A}}^{-}P_{A}^{\perp }$, with $P^{\perp }_{A}=I_{m}-AA^{+}$, ${\bar{A}}=P^{\perp }_{C}A$, $P^{\perp }_{C}=I_{m}-CC^{+}$ with $C^{+}=(C^{T}Q_{yy}^{-1}C)^{-1}C^{T}Q_{yy}^{-1}$, ${\bar{A}}^{-}=S\;[({\bar{A}}S)^{T}Q_{yy}^{-1}({\bar{A}}S)]^{-1}({\bar{A}}S)^{T}Q_{yy}^{-1}$, with *S* a basis matrix of which the range space is complementary to that of *V*.

#### Proof

See the Appendix. $\square$

Note if one opts for the adaptation of ${\hat{\theta }}_{0}$ as given above, that one cannot use the expression for the DIA estimator as given in (13) anymore. For example, if the hypotheses ${\mathcal {H}}_{i}$, with $i=1,\cdots ,l$, are indistinguishable, i.e., ${\mathcal {P}}_{1}=\ldots ={\mathcal {P}}_{l}$, the adaptation according to (26) implies that the DIA estimator in (13) changes to

27 $$\begin{aligned} {\bar{\theta }}\;=\;{\hat{\theta }}_{0}\,{p}_{0}(t)+{\hat{\theta }}\,{p}_{1}(t)+\sum \limits _{j=l+1}^{k}\;{\hat{\theta }}_{j}\,{p}_{j}(t) \end{aligned}$$

Thus, the $k+1$ terms in the sum are now reduced to $k-l+2$, with ${\hat{\theta }}$ being the BLUE under (24).

## Numerical analysis

In this section, we apply the theory of the previous sections to some selected examples so as to illustrate and explain the performance of the various decision elements in DIA-datasnooping. The insight so obtained will also help us appreciate some of the more complex intricacies of the theory. The following three different cases are considered: height-difference observations of a leveling network, distance measurements of a horizontal geodetic network and pseudorange measurements between a single ground station and GPS satellites. We analyze and illustrate how geometry changes in the measurement setup affect the testing procedure, including its partitioning of the misclosure space, and the corresponding CD probabilities (MDB) and CI probabilities (MIB). The CD probability under ${\mathcal {H}}_{i}$ ($i=1,\ldots ,k$) is computed based on (16) from $\chi ^{2}(r,\lambda _{i}^{2})$, whereas the CI probability under ${\mathcal {H}}_{i}$ ($i=1,\ldots ,k$) is computed as described in the Appendix.

### Leveling network

**Fig. 1:**
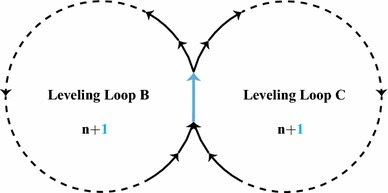
A leveling network consisting of two leveling loops with *n* observations each and one shared observation (blue)

Suppose that we have two leveling loops containing $n\ge 2$ height-difference observations each and sharing one observation with each other (see Fig. 1). For such leveling network, two misclosures can be formed stating that the sum of observations in each loop equals zero. Assuming that all the observations are uncorrelated and of the same precision $\sigma$, a misclosure vector *t* and its variance matrix $Q_{tt}$ can be formed as28where $y_{\mathsf {A}}$ is the observation shared between the two leveling loops, and $y_{\mathsf {B}}$ and $y_{\mathsf {C}}$ the *n*-vectors of observations of the leveling loops $\mathsf {B}$ and $\mathsf {C}$, respectively. The number of datasnooping alternative hypotheses for the above model is equal to $2n+1$. But it will be clear of course that not all of them are separately identifiable. Looking at the structure of $B^{T}$ in (28), it can be seen that out of $2n+1$ vectors $c_{t_{i}}$ (columns of $B^{T}$), only the following three are nonparallel

29 $$\begin{aligned} \begin{array}{l} c_{t_{\mathsf {A}}}=\left[ \begin{array}{c}1 \\ 1\end{array}\right] ,~ c_{t_{\mathsf {B}}}=\left[ \begin{array}{c}1 \\ 0\end{array}\right] ,~ c_{t_{\mathsf {C}}}=\left[ \begin{array}{c}0 \\ 1\end{array}\right] \end{array} \end{aligned}$$

which implies that in each leveling loop excluding the shared observation $y_{\mathsf {A}}$, an outlier on each of the observations is sensed in the same way by the vector of misclosures. In other words, the testing procedure cannot distinguish between the outliers on the observations in $y_{\mathsf {B}}$, and between those on the observations in $y_{\mathsf {C}}$. Therefore, among the $2n+1$ alternative hypotheses, we retain three: ${\mathcal {H}}_{\mathsf {A}}$ corresponding with $y_{\mathsf {A}}$, ${\mathcal {H}}_{\mathsf {B}}$ corresponding with one of the observations in $y_{\mathsf {B}}$ and ${\mathcal {H}}_{\mathsf {C}}$ corresponding with one of the observations in $y_{\mathsf {C}}$.

**Fig. 2:**
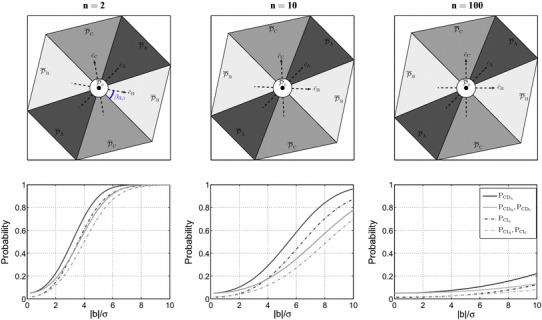
Visualization of the datasnooping testing procedure defined in Sect. 2.2 for the leveling network shown in Fig. 1 assuming $\alpha =0.05$ and $\sigma =5$ mm. [Top] Datasnooping partitioning of the misclosure space ${\mathbb {R}}^{2}$ corresponding with ${\bar{t}}$ (cf. 9). [Bottom] The graphs of CD (solid lines) and CI probability (dashed lines) of different alternative hypotheses as function of bias-to-noise ratio

#### Misclosure space partitioning

Given (29), the datasnooping partitioning of the misclosure space is formed by *four* distinct regions ${\mathcal {P}}_{i}$ with $i\in \{0,\mathsf {A},\mathsf {B},\mathsf {C}\}$. For the sake of visualization, instead of *t*, we work with ${\bar{t}}$ (cf. 9). The datasnooping partitioning, as mentioned earlier, is then driven by the relative orientation of ${\bar{c}}_{\mathsf {A}}$, ${\bar{c}}_{\mathsf {B}}$ and ${\bar{c}}_{\mathsf {C}}$ (cf. 11). The angles between these unit vectors are computed as

30 $$\begin{aligned} \angle ({\bar{c}}_{\mathsf {A}},{\bar{c}}_{\mathsf {B}})= & {} \angle ({\bar{c}}_{\mathsf {A}},{\bar{c}}_{\mathsf {C}})=\cos ^{-1} \sqrt{\dfrac{n}{2(n+1)}}\nonumber \\ \angle ({\bar{c}}_{\mathsf {B}},{\bar{c}}_{\mathsf {C}})= & {} \cos ^{-1} {\dfrac{-1}{n+1}} \end{aligned}$$

As (30) suggests, when $n\rightarrow \infty$, the angles $\angle ({\bar{c}}_{\mathsf {A}},{\bar{c}}_{\mathsf {B}})$ and $\angle ({\bar{c}}_{\mathsf {A}},{\bar{c}}_{\mathsf {C}})$ go to $45^{\circ }$, and the angle $\angle ({\bar{c}}_{\mathsf {B}},{\bar{c}}_{\mathsf {C}})$ goes to $90^{\circ }$. Figure 2 demonstrates the impact of *n* on the misclosure space partitioning given $\alpha =0.05$, $r=2$ and $\sigma =5$ mm. Using different shades of *gray* color, the *first row* of Fig. 2 shows, for $n=2$, $n=10$ and $n=100$, the partitioning of the misclosure space formed by $\overline{{\mathcal {P}}}_{i}$ with $i\in \{0,\mathsf {A},\mathsf {B},\mathsf {C}\}$.

#### CD and CI probabilities

According to (17), for a given $\lambda (\alpha ,\gamma _{\mathrm{CD}},r)$, the MDB depends only on $\Vert c_{t_{i}}\Vert _{Q_{tt}}$. For the leveling network characterized in (28) and its corresponding vectors $c_{t_{i}}$ in (29), we have

31 $$\begin{aligned} \Vert c_{t_{\mathsf {A}}}\Vert _{Q_{tt}}= & {} \sigma ^{-1}\sqrt{\dfrac{2}{n+2}} \nonumber \\ \Vert c_{t_{\mathsf {B}}}\Vert _{Q_{tt}}= & {} \Vert c_{t_{\mathsf {C}}}\Vert _{Q_{tt}}=\sigma ^{-1}\sqrt{\dfrac{n+1}{n(n+2)}} \end{aligned}$$

which clearly shows that for a given set of $\{\alpha ,\gamma _{\mathrm{CD}},r\}$, smaller ${\mathcal {H}}_{\mathsf {A}}$-biases can be detected compared to ${\mathcal {H}}_{\mathsf {B}}$ and ${\mathcal {H}}_{\mathsf {C}}$. Equivalently, it can be stated that for a given $\{\alpha ,r\}$ and $b_{i}=b$, the CD probability of ${\mathcal {H}}_{\mathsf {A}}$ is larger than that of ${\mathcal {H}}_{\mathsf {B}}$ and ${\mathcal {H}}_{\mathsf {C}}$. That is because each observation in $y_{\mathsf {B}}$ and $y_{\mathsf {C}}$ contributes to only one leveling loop while $y_{\mathsf {A}}$ contributes to two leveling loops, thus being checked by the observations of both loops. The solid curves in Fig. 2 (second row) depict $\mathrm{P}_{\mathrm{CD}_{i}}$ as function of the bias-to-noise ratio $|b|/\sigma$. The *dark gray* graphs correspond with ${\mathcal {H}}_{\mathsf {A}}$, while the *light gray* graphs correspond with ${\mathcal {H}}_{\mathsf {B}}$ and ${\mathcal {H}}_{\mathsf {C}}$. These graphs can be used as follows. For a certain $b_{i}=b$, one can compare the corresponding $\mathrm{P}_{\mathrm{CD}_{i}}$ of different alternative hypotheses. One can also take the reverse route by comparing the MDB of different alternative hypotheses for a certain $\mathrm{P}_{\mathrm{CD}_{i}}=\gamma _{\mathrm{CD}}$. In agreement with (31), the solid *dark gray* graphs always lie above the solid *light gray* ones. As the number of observations increases in each loop ($n\uparrow$), the corresponding $\mathrm{P}_{\mathrm{CD}_{i}}$ decreases for a given ${\mathcal {H}}_{i}$-bias. This is due to the fact that the variance of the misclosure vector is an increasing function of the number of observations in each loop (see 28). The lower the precision of the misclosures, the lower is the sensitivity of the testing procedure to a given bias in an observation.

**Fig. 3:**
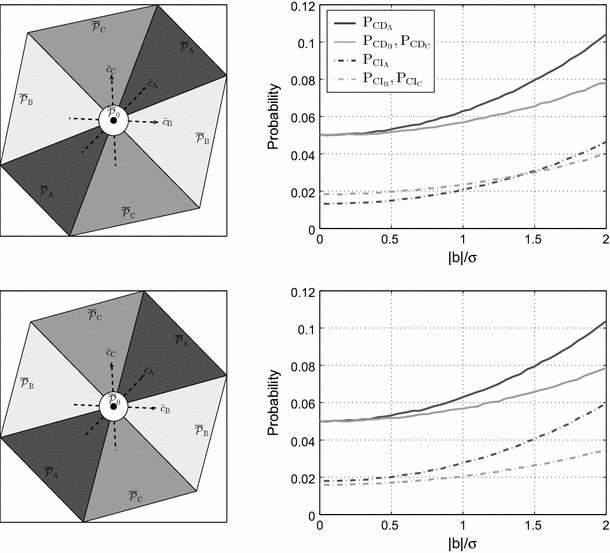
Comparing two testing scheme for the leveling network in Fig. 1 assuming $n=10$, $\alpha =0.05$ and $\sigma =5$ mm. [Top] Datasnooping testing procedure defined in Sect. 2.2. [Bottom] The testing procedure defined by (32). [Left] Partitioning of the misclosure space corresponding with ${\bar{t}}$. [Right] The graphs of CD (solid lines) and CI probabilities (dashed lines) of different alternative hypotheses as function of bias-to-noise ratio

The dashed curves in Fig. 2 (second row) depict $\mathrm{P}_{\mathrm{CI}_{i}}$ as function of $|b|/\sigma$. These curves ($\mathrm{P}_{\mathrm{CI}_{i}}$) always lie below their solid counterparts ($\mathrm{P}_{\mathrm{CD}_{i}}$). Like the solid graphs, these dashed graphs can be used either for comparing the MIB of different alternative hypotheses given a certain $\mathrm{P}_{\mathrm{CI}_{i}}=\gamma _{\mathrm{CI}}$, or for comparing the corresponding $\mathrm{P}_{\mathrm{CI}_{i}}$ of different alternative hypotheses given a certain $b_{i}=b$. We note that despite the CD probability of ${\mathcal {H}}_{\mathsf {A}}$ being *always* larger than that of ${\mathcal {H}}_{\mathsf {B}}$ and ${\mathcal {H}}_{\mathsf {C}}$, the CI probability of ${\mathcal {H}}_{\mathsf {A}}$ is *not always* larger than that of ${\mathcal {H}}_{\mathsf {B}}$ and ${\mathcal {H}}_{\mathsf {C}}$. Depending on the number of measurements in each loop *n*, if $|b|/\sigma$ is smaller than a certain value, then we have $\mathrm{P}_{\mathrm{CI}_{\mathsf {A}}}<\mathrm{P}_{\mathrm{CI}_{\mathsf {B}}}=\mathrm{P}_{\mathrm{CI}_{\mathsf {C}}}$. This discrepancy between the behavior of CD probability and that of CI probability as function of $|b|/\sigma$ for a given $\alpha$ is due to the fact that while $\mathrm{P}_{\mathrm{CD}_{i}}$ is driven only by $\Vert c_{t_{i}}\Vert _{Q_{tt}}$, $\mathrm{P}_{\mathrm{CI}_{i}}$ is in addition driven by $\overline{{\mathcal {P}}}_{i}$ and the orientation of ${\bar{c}}_{i}$ w.r.t. the straight borders of $\overline{{\mathcal {P}}}_{i}$ (cf. 19). Looking at the first row of Fig. 2, we note that $\overline{{\mathcal {P}}}_{\mathsf {A}}$ has smaller area compared to $\overline{{\mathcal {P}}}_{\mathsf {B}}$ and $\overline{{\mathcal {P}}}_{\mathsf {C}}$. Therefore, |*b*| should be large enough such that $\Vert c_{t_{\mathsf {A}}}\Vert _{Q_{tt}}>\Vert c_{t_{\mathsf {B}}}\Vert _{Q_{tt}}=\Vert c_{t_{\mathsf {C}}}\Vert _{Q_{tt}}$ can compensate for $\overline{{\mathcal {P}}}_{\mathsf {A}}$ being smaller than $\overline{{\mathcal {P}}}_{\mathsf {B}}$ and $\overline{{\mathcal {P}}}_{\mathsf {C}}$.

#### Impact of partitioning on CI probability

As was mentioned, $\mathrm{P}_{\mathrm{CI}_{i}}$ depends on $\overline{{\mathcal {P}}}_{i}$, the orientation of $\bar{c_{i}}$ and the magnitude of $\Vert c_{t_{i}}\Vert _{Q_{tt}}$. While the last two factors are driven by the underlying model, the first one depends on the testing procedure. Our above conclusions about the CI probability will then change if we go for another testing scheme. For example, let $\overline{{\mathcal {P}}}_{0}$ be defined by (10) and

32 $$\begin{aligned} \overline{{\mathcal {P}}}_{i\ne 0}=\left\{ {\bar{t}}\in {\mathbb {R}}^{2}/\overline{{\mathcal {P}}}_{0}|~|d_{i}^{T}{\bar{t}}|=\underset{k\in \{\mathsf {A},\mathsf {B},\mathsf {C}\}}{\max }\;|d_{k}^{T}{\bar{t}}|\right\} \end{aligned}$$

where $d_{\mathsf {A}}={\bar{c}}_{\mathsf {A}}$, $d_{\mathsf {B}}=R_{(-60^{\circ })}{\bar{c}}_{\mathsf {A}}$ and $d_{\mathsf {C}}=R_{(60^{\circ })}{\bar{c}}_{\mathsf {A}}$ with $R_{\theta }$ being the counterclockwise rotation matrix. This testing scheme leads to $\overline{{\mathcal {P}}}_{\mathsf {A}}$, $\overline{{\mathcal {P}}}_{\mathsf {B}}$ and $\overline{{\mathcal {P}}}_{\mathsf {C}}$ to be of the same shape. In addition, while ${\bar{c}}_{{\mathsf {A}}}$ is parallel to the bisector line of the angle between the two straight borders of $\overline{{\mathcal {P}}}_{\mathsf {A}}$, ${\bar{c}}_{{\mathsf {B}}}$ and ${\bar{c}}_{{\mathsf {C}}}$ are close to one of the straight borders of their corresponding region. This combined with the fact that $\Vert c_{t_{\mathsf {A}}}\Vert _{Q_{tt}}>\Vert c_{t_{\mathsf {B}}}\Vert _{Q_{tt}}=\Vert c_{t_{\mathsf {C}}}\Vert _{Q_{tt}}$ lead us to the conclusion that $P_{\mathrm{CI}_{\mathsf {A}}}>P_{\mathrm{CI}_{\mathsf {B}}}=P_{\mathrm{CI}_{\mathsf {C}}}$ holds for any given bias *b*. Figure 3 shows the difference between the testing procedure based on (11) and (32), in terms of misclosure space partitioning [left] and CD and CI probability [right].

### Horizontal geodetic network

Consider a horizontal geodetic network containing *m* reference points from which we measure distances toward an unknown point to determine its horizontal coordinates. Assuming that all the measurements are uncorrelated and of the same precision, the design matrix and the observations variance matrix of the linearized model under ${\mathcal {H}}_{0}$ read

33 $$\begin{aligned} A\; =\; \left[ \begin{array}{c} -\,u_{1}^{T}\\ \vdots \\ -\,u_{m}^{T} \end{array}\right] \qquad ,\qquad Q_{yy} = \sigma ^{2}\,I_{m} \end{aligned}$$

where the unit direction 2-vector from the unknown point to the reference point $(i=1,\ldots ,m)$ is denoted by $u_{i}$. In this observational model, the redundancy is $r=m-2$ revealing that the misclosure vector *t* is of dimension $m-2$.

#### Misclosure space partitioning

**Fig. 4:**
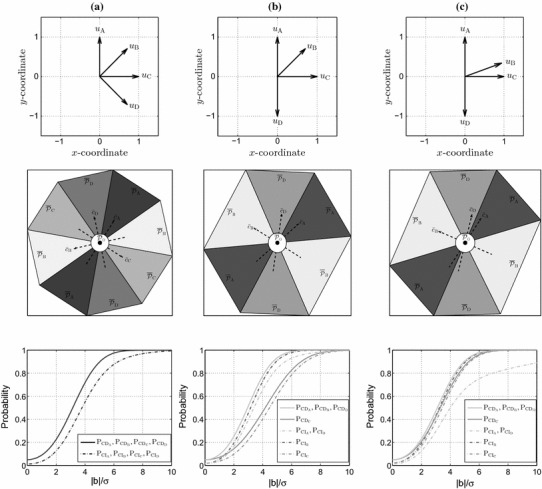
Visualization of the datasnooping testing procedure defined in Sect. 2.2 for the horizontal geodetic networks shown in the first row assuming $\alpha =0.05$ and $\sigma =5$ mm. [Top] Geometry of the four reference points w.r.t. the point of which the coordinates are to be estimated. [Middle] Datasnooping partitioning of the misclosure space ${\mathbb {R}}^{2}$ corresponding with ${\bar{t}}$ (cf. 9). [Bottom] The graphs of CD (solid lines) and CI probability (dashed lines) of different alternative hypotheses as function of bias-to-noise ratio

For the model in (33), the angles between the corresponding ${\bar{c}}_{{i}}$ vectors are computed as

34 $$\begin{aligned} \cos \angle ({\bar{c}}_{{i}},{\bar{c}}_{{j}})= & {} \dfrac{-\,u_{i}^{T}C^{-1}_{xx}u_{j}}{\sqrt{(1-\Vert u_{i}\Vert ^{2}_{C_{xx}})\times (1-\Vert u_{j}\Vert ^{2}_{C_{xx}})}} \end{aligned}$$

which is a consequence of $BQ_{tt}^{-1}B^{T}=Q_{yy}^{-1}-Q_{yy}^{-1}AQ_{xx}A^{T}Q_{yy}^{-1}$ with $Q_{xx}=\sigma ^{2}\,C_{xx}^{-1}$ and $C_{xx}=\sum _{k=1}^{m}u_{k}u_{k}^{T}$. Assuming that the horizontal geodetic network comprises $m=4$ reference points, Fig. 4 presents the same information as Fig. 2 but for geodetic networks corresponding with (33). The first row shows the orientation of vectors $u_{i}$. The standard deviation of the distance measurements is considered to be $\sigma =5$ mm, and the false alarm is set to $\alpha =0.05$. In (a), the geometry of the measuring points leads to a cofactor matrix of $C_{xx}=2\,I_{2}$ of which the substitution in (34) gives $\cos \angle ({\bar{c}}_{{i}},{\bar{c}}_{{j}})=-\cos \angle (u_{{i}},u_{{j}})$. Given that the angle between consecutive vectors $u_{i}$ is $45^{\circ }$, the four regions $\overline{{\mathcal {P}}}_{i\ne 0}$ have then the same shape. Moving the reference point $\mathsf {D}$ to a new location such that $u_{\mathsf {D}}=-u_{\mathsf {A}}$ as illustrated in (b), the two regions $\overline{{\mathcal {P}}}_{\mathsf {B}}$ and $\overline{{\mathcal {P}}}_{\mathsf {C}}$, as Theorem 1 states, become identical. The proof is given as follows. Let $u_{\mathsf {D}}=p\,u_{\mathsf {A}}$ ($p=\pm 1$). As the vectors $c_{t_{i}}$ are the columns of $B^{T}$ and given that $B^{T}A=0$, we have

35 $$\begin{aligned} (c_{t_\mathsf {A}}+p\,c_{t_\mathsf {D}})u_{\mathsf {A}}^{T}+c_{t_\mathsf {B}}u_{\mathsf {B}}^{T}+c_{t_\mathsf {C}}u_{\mathsf {C}}^{T}=0 \end{aligned}$$

Multiplying both sides of the above equation with $u_{\mathsf {A}}^{\perp }$ from the right, we get

36 $$\begin{aligned} c_{t_\mathsf {C}}=-\,\dfrac{u_{\mathsf {B}}^{T}u_{\mathsf {A}}^{\perp }}{u_{\mathsf {C}}^{T}u_{\mathsf {A}}^{\perp }}c_{t_\mathsf {B}} \end{aligned}$$

which means that $c_{t_\mathsf {B}}\parallel c_{t_\mathsf {C}}$, thus ${\bar{c}}_{\mathsf {B}}\parallel {\bar{c}}_{\mathsf {C}}$ and $\overline{{\mathcal {P}}}_{\mathsf {B}}=\overline{{\mathcal {P}}}_{\mathsf {C}}$. If in addition, we have $u_{\mathsf {C}}=q \,u_{\mathsf {B}}$ ($q=\pm 1$), then (35) simplifies to

37 $$\begin{aligned} (c_{t_\mathsf {A}}+p\,c_{t_\mathsf {D}})u_{\mathsf {A}}^{T}+(c_{t_\mathsf {B}}+q\,c_{t_\mathsf {C}})u_{\mathsf {B}}^{T}=0 \end{aligned}$$

Multiplying the above once with $u_{\mathsf {A}}^{\perp }$ and once with $u_{\mathsf {B}}^{\perp }$ from the right, then we get $c_{t_\mathsf {A}}\parallel c_{t_\mathsf {D}}$ and $c_{t_\mathsf {B}}\parallel c_{t_\mathsf {C}}$, thus ${\bar{c}}_{\mathsf {A}}\parallel {\bar{c}}_{\mathsf {D}}$ and ${\bar{c}}_{\mathsf {B}}\parallel {\bar{c}}_{\mathsf {C}}$. From (b) to (c), as the angle between $u_{\mathsf {B}}$ and $u_{\mathsf {C}}$ decreases, the errors in the measurements $\mathsf {A}$ and $\mathsf {D}$ become less distinguishable from each other, but better separable from those in the measurements of $\mathsf {B}$ and $\mathsf {C}$.

#### CD and CI probabilities

The illustrations on the third row of Fig. 4 show the graphs of $\mathrm{P}_{\mathrm{CD}_{i}}$ (solid lines) and $\mathrm{P}_{\mathrm{CI}_{i}}$ (dashed lines) under all the four alternative hypotheses ${\mathcal {H}}_{i}$ with $i\in \{\mathsf {A},\mathsf {B},\mathsf {C},\mathsf {D}\}$. The CD probability $\mathrm{P}_{\mathrm{CD}_{i}}$ corresponding with (33) for a given $\alpha$, *r* and a bias value |*b*| is driven by (cf. 17)

38 $$\begin{aligned} \Vert c_{t_{i}}\Vert _{Q_{tt}}= & {} \sigma ^{-1}\,\left[ 1-\dfrac{1}{\mathrm{det}(C_{xx})}\sum \limits _{j=1}^{m}\;\sin ^{2}\angle (u_{i},u_{j})\right] ^{\frac{1}{2}} \end{aligned}$$

with det(.) being the determinant operator. In (a), owing to $45^{\circ }$ angle between the consecutive vectors $u_{i}$, we have $\Vert c_{t_{i}}\Vert _{Q_{tt}}=\Vert c_{t_{j}}\Vert _{Q_{tt}}$ for any $i\ne j$, hence $\mathrm{P}_{\mathrm{CD}_{i}}=\mathrm{P}_{\mathrm{CD}_{j}}$ for any given value of bias |*b*| and $i\ne j$. Furthermore, as a consequence of having a symmetric partitioning, we also have $\mathrm{P}_{\mathrm{CI}_{i}}=\mathrm{P}_{\mathrm{CI}_{j}}$ for any given value of bias |*b*| and $i\ne j$. In (b) and (c), given that $u_{\mathsf {A}}\parallel u_{\mathsf {D}}$ and $u_{\mathsf {A}}\perp u_{\mathsf {C}}$, we have $\Vert c_{t_{\mathsf {A}}}\Vert _{Q_{tt}}=\Vert c_{t_{\mathsf {B}}}\Vert _{Q_{tt}}=\Vert c_{t_{\mathsf {D}}}\Vert _{Q_{tt}}$ conveying that the hypotheses ${\mathcal {H}}_{\mathsf {A}}$, ${\mathcal {H}}_{\mathsf {B}}$ and ${\mathcal {H}}_{\mathsf {D}}$ have the same CD probability. ${\mathcal {H}}_{\mathsf {A}}$ and ${\mathcal {H}}_{\mathsf {D}}$ have, in addition, the same CI probability since $\overline{{\mathcal {P}}}_{\mathsf {A}}$ and $\overline{{\mathcal {P}}}_{\mathsf {D}}$ have the same shape and also the orientation of ${\bar{c}}_{{\mathsf {A}}}$ inside $\overline{{\mathcal {P}}}_{\mathsf {A}}$ is the same as that of ${\bar{c}}_{{\mathsf {D}}}$ inside $\overline{{\mathcal {P}}}_{\mathsf {D}}$.

In (b) and (c), ${\mathcal {H}}_{\mathsf {B}}$ is not distinguishable from ${\mathcal {H}}_{\mathsf {C}}$. For these hypotheses, although not identifiable from each other, we still define CI probability as $\mathrm{P}_{\mathrm{CI}_{\mathsf {B}}}=\mathrm{P}({\bar{t}}\in \overline{{\mathcal {P}}}_{\mathsf {B}}|{\mathcal {H}}_{\mathsf {B}})$ and $\mathrm{P}_{\mathrm{CI}_{\mathsf {C}}}=\mathrm{P}({\bar{t}}\in \overline{{\mathcal {P}}}_{\mathsf {B}}|{\mathcal {H}}_{\mathsf {C}})$. It can be seen that, although ${\mathcal {H}}_{\mathsf {B}}$ is not distinguishable from ${\mathcal {H}}_{\mathsf {C}}$, they are different in both the CD and CI probabilities. Also, the testing procedure is more sensitive to the biases in $y_{\mathsf {B}}$ compared to the same biases in $y_{\mathsf {C}}$. This is due to the fact that the observation of $\mathsf {C}$ contributes to the misclosure vector less than the observation of $\mathsf {B}$. The contribution of the measurement of $\mathsf {C}$ to the misclosure vector depends on the relative orientation of $u_{\mathsf {B}}$ w.r.t. $u_{\mathsf {C}}$. In case $u_{\mathsf {B}}$ is parallel to $u_{\mathsf {A}}$ and $u_{\mathsf {D}}$, the measurement of the point $\mathsf {C}$ would have zero contribution to the misclosure vector and cannot be screened at all. As the angle between $u_{\mathsf {B}}$ and $u_{\mathsf {C}}$ decreases, the mentioned contribution increases, so does the sensitivity of the testing procedure to the biases in the measurement of $\mathsf {C}$.

Note that for the geometries shown in (b) and (c), if the misclosure vector lies in $\overline{{\mathcal {P}}}_{\mathsf {B}}$, it cannot be inferred that whether $y_{\mathsf {B}}$ or $y_{\mathsf {C}}$ is biased. For adaptation, one may extend the design matrix *A* to $[A~~c_{\mathsf {B}}~~c_{\mathsf {C}}]$, which would be of relevance if the parameters of interest remain estimable (see Theorem 2). As $c_{\mathsf {B}}$ and $c_{\mathsf {C}}$ are canonical unit vectors, then $[c_{\mathsf {B}}~~c_{\mathsf {C}}]^{\perp ^{T}}A$ is a matrix achieved by removing the rows of *A* corresponding with $y_{\mathsf {B}}$ and $y_{\mathsf {C}}$ as39which clearly shows that the *x*-coordinate is not estimable. However, the above adaptation strategy is still of relevance if one is interested in the *y*-coordinate.

A summary of the above qualitative findings in relation to the geometry of the measuring points is given as follows

- If $|\cos \angle (u_{i},u_{i+1})|=\cos 45^{\circ }$ for any $i=1,2,3$, then
  - $$|\cos \angle ({\bar{c}}_{i},{\bar{c}}_{i+1})|=\cos 45^{\circ }$$
  - $\overline{{\mathcal {P}}}_{i}$ has the same shape of $\overline{{\mathcal {P}}}_{j}$ for any $i\ne j$
  - $\mathrm{P}_{\mathrm{CD}_{i}}=\mathrm{P}_{\mathrm{CD}_{j}}$ and $\mathrm{P}_{\mathrm{CI}_{i}}=\mathrm{P}_{\mathrm{CI}_{j}}$ for any $i\ne j$
- If $$u_{\mathsf {A}}\parallel u_{\mathsf {D}}$$, then
  - $$\overline{{\mathcal {P}}}_{\mathsf {B}}=\overline{{\mathcal {P}}}_{\mathsf {C}}$$
  - $$\mathrm{P}_{\mathrm{CD}_{\mathsf {A}}}=\mathrm{P}_{\mathrm{CD}_{\mathsf {D}}}$$ and $$\mathrm{P}_{\mathrm{CI}_{\mathsf {A}}}=\mathrm{P}_{\mathrm{CI}_{\mathsf {D}}}$$
- If $u_{\mathsf {A}}\parallel u_{\mathsf {D}}$ and $u_{\mathsf {B}}\parallel u_{\mathsf {C}}$, then
  - $$\overline{{\mathcal {P}}}_{\mathsf {A}}=\overline{{\mathcal {P}}}_{\mathsf {D}}$$ and $$\overline{{\mathcal {P}}}_{\mathsf {B}}=\overline{{\mathcal {P}}}_{\mathsf {C}}$$
  - $\overline{{\mathcal {P}}}_{\mathsf {A}}$ has the same shape of $\overline{{\mathcal {P}}}_{\mathsf {B}}$
  - $\mathrm{P}_{\mathrm{CD}_{i}}=\mathrm{P}_{\mathrm{CD}_{j}}$ and $\mathrm{P}_{\mathrm{CI}_{i}}=\mathrm{P}_{\mathrm{CI}_{j}}$ for any $i\ne j$
- If $u_{\mathsf {A}}\parallel u_{\mathsf {D}}$ and $u_{\mathsf {C}}\perp u_{\mathsf {A}}$, then
  - $$\mathrm{P}_{\mathrm{CD}_{\mathsf {A}}}=\mathrm{P}_{\mathrm{CD}_{\mathsf {B}}}=\mathrm{P}_{\mathrm{CD}_{\mathsf {D}}}$$
  - $$\mathrm{P}_{\mathrm{CD}_{\mathsf {B}}}\ge \mathrm{P}_{\mathrm{CD}_{\mathsf {C}}}$$ and $$\mathrm{P}_{\mathrm{CI}_{\mathsf {B}}}\ge \mathrm{P}_{\mathrm{CI}_{\mathsf {C}}}$$.
  - If $\angle (u_{\mathsf {B}},u_{\mathsf {C}})$ decreases, so does the differences $\mathrm{P}_{\mathrm{CD}_{\mathsf {B}}}-\mathrm{P}_{\mathrm{CD}_{\mathsf {C}}}$ and $\mathrm{P}_{\mathrm{CI}_{\mathsf {B}}}-\mathrm{P}_{\mathrm{CI}_{\mathsf {C}}}$.
- If $u_{\mathsf {A}}\parallel u_{\mathsf {B}}$, $u_{\mathsf {A}}\parallel u_{\mathsf {D}}$ and $u_{\mathsf {C}}\perp u_{\mathsf {A}}$, then $\mathrm{P}_{\mathrm{CD}_{\mathsf {C}}}=\mathrm{P}_{\mathrm{CI}_{\mathsf {C}}}=0$.

### GPS single-point positioning

Let the pseudorange observations of *m* GPS satellites be collected by one single receiver to estimate its three-dimensional position coordinates and clock error. Assuming that all the code observations are uncorrelated and of the same precision $\sigma$, the corresponding linearized observational model, also known as the single-point positioning (SPP) model, under ${\mathcal {H}}_{0}$ is characterized through the following full-rank design matrix and the observations variance matrix40in which the 3-vectors $u_{i}$ ($i=1,\ldots ,m$) are the receiver-satellite unit direction vectors. The first three columns of *A* correspond with the receiver North-East-Up coordinate increments while the last one corresponds with the receiver clock error increment. Given that the design matrix *A* is of order $m\times 4$, the redundancy of the SPP model is $r=m-4$.

#### Misclosure space partitioning

**Fig. 5:**
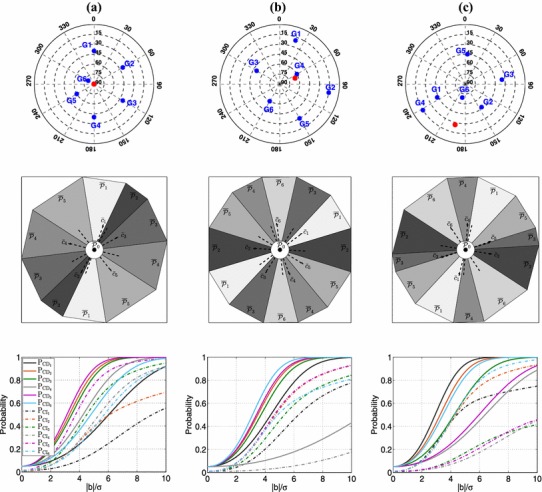
Visualization of the datasnooping testing procedure defined in Sect. 2.2 for the SPP assuming $\alpha =0.05$ and $\sigma =30$ cm. [Top] Skyplot views of the satellite geometries. The six blue circles in each panel denote the skyplot position of the satellites. The red circle denotes the skyplot position of the symmetry axis of the cone formed by the satellites G*i* with $i=1,2,3,4$ in **a**, $i=1,2,3,5,6$ in **b** and $i=1,2,4,6$ in **c**. [Middle] Datasnooping partitioning of the misclosure space ${\mathbb {R}}^{2}$ corresponding with ${\bar{t}}$ (cf. 9). [Bottom] The graphs of CD (solid lines) and CI probabilities (dashed lines) of different alternative hypotheses as function of bias-to-noise ratio

With the SPP model in (40), the angles between the vectors ${\bar{c}}_{{i}}$ are computed as41in which $C_{xx}=\sum _{k=1}^{m}(u_{k}-{\bar{u}})(u_{k}-{\bar{u}})^{T}$ and ${\bar{u}}=\frac{1}{m}\sum _{k=1}^{m}u_{k}$. Assuming that six GPS satellites are transmitting signals to a single receiver ($m=6$), two misclosures can be formed, i.e., $r=2$. Figure 5, for three different geometries of these satellites (first row), shows the partitioning of the misclosure space (second row). The satellite geometries in (a) and (b) are artificial while that in (c), except for the name of satellites, is a real GPS geometry at Perth, Australia.

In (a), despite having six pseudorange observations, the partitioning is formed by five distinct regions. The regions corresponding with ${\mathcal {H}}_{5}$ and ${\mathcal {H}}_{6}$ coincide each other, i.e., $\overline{{\mathcal {P}}}_{5}=\overline{{\mathcal {P}}}_{6}$, which can be explained as follows. The lines-of-sight of the four satellites G1, G2, G3, and G4 lie on a cone of which the symmetry axis is indicated as the *red* circle. Therefore, we have

42 $$\begin{aligned} u_{i}^{T}\,d\;=\;c;\qquad i=1,\ldots ,4 \end{aligned}$$

with *d* the unit 3-vector of the symmetry axis of the mentioned cone and *c* the cosine of the half the vertex angle of the cone. The extended SPP design matrix $[A~~ c_{5}~~ c_{6}]$ will then satisfy

43 $$\begin{aligned}{}[A~~ c_{5}~~ c_{6}]\;\left[ \begin{array}{c}d\\ c\\ u_{5}^{T}d-c\\[1mm]u_{6}^{T}d-c \end{array}\right] =0 \end{aligned}$$

Therefore, the $6\times 6$ matrix $[A~~ c_{5}~~ c_{6}]$ is rank-deficient which, according to Theorem 1, implies that the two alternative hypotheses ${\mathcal {H}}_{5}$ and ${\mathcal {H}}_{6}$ are not separable. If the misclosure vector lies in $\overline{{\mathcal {P}}}_{5}$, it cannot be inferred that whether observation $y_{5}$ or $y_{6}$ is biased. For adaptation, one may use the above-extended design matrix in case the parameters of interest remain estimable (see Theorem 2). As $c_{5}$ and $c_{6}$ are canonical unit vectors, then $[c_{5}~~c_{6}]^{\perp ^{T}}A$ is a matrix achieved by removing the last two rows of *A*. Based on such reduced design matrix, according to (42), the position solution in the direction of *d* is indeterminate. Since *d* is vertically oriented, the horizontal coordinates (East-North) remain estimable based on the first four rows of *A*.

In (b), all the alternative hypotheses are distinguishable. In (c), the two vectors ${\bar{c}}_{3}$ and ${\bar{c}}_{5}$ are almost parallel which is due to the satellites G1, G2, G4 and G6 forming a cone-like geometry of which the axis is indicated by a *red* circle.

#### CD and CI probabilities

The graphs of $\mathrm{P}_{\mathrm{CD}_{i}}$ and $\mathrm{P}_{\mathrm{CI}_{i}}$ for $i=1,\ldots ,6$ as function of the bias-to-noise ratio are given in the third row of Fig. 5. One notes that the signature of $\mathrm{P}_{\mathrm{CD}_{i}}$ is generally different from $\mathrm{P}_{\mathrm{CI}_{i}}$. For example, in (a), we have $\mathrm{P}_{\mathrm{CD}_{2}}>\mathrm{P}_{\mathrm{CD}_{3}}$ while $\mathrm{P}_{\mathrm{CI}_{3}}>\mathrm{P}_{\mathrm{CI}_{2}}$. That is because $\mathrm{P}_{\mathrm{CI}_{i}}$, in addition to $\Vert c_{t_i}\Vert _{Q_{tt}}$, is also driven by $\overline{{\mathcal {P}}}_{i}$ and the orientation of ${\bar{c}}_{i}$ within $\overline{{\mathcal {P}}}_{i}$. In (a), we also note that although ${\mathcal {H}}_{5}$ and ${\mathcal {H}}_{6}$ cannot be distinguished, the testing procedure has a different sensitivity to the ${\mathcal {H}}_{5}$- and ${\mathcal {H}}_{6}$-biases. For the same bias-to-noise ratios, we have $\mathrm{P}_{\mathrm{CD}_{5}}>\mathrm{P}_{\mathrm{CD}_{6}}$ and $\mathrm{P}_{\mathrm{CI}_{5}}>\mathrm{P}_{\mathrm{CI}_{6}}$, which can be explained as follows. The difference between $\mathrm{P}_{\mathrm{CD}_{5}}$ and $\mathrm{P}_{\mathrm{CD}_{6}}$ for a given bias-to-noise ratio lies in the difference between $\Vert c_{t_{5}}\Vert _{Q_{tt}}$ and $\Vert c_{t_{6}}\Vert _{Q_{tt}}$ (cf. 17). Given that $c_{t_{i}}$ is the *i*th column of $B^{T}$ and given (42), multiplying the corresponding SPP design matrix *A* with $B^{T}$ from left and with $[d^{T},~c]^{T}$ from right, we arrive at

44 $$\begin{aligned} c_{t_{5}}\;=\;-\dfrac{u_{6}^{T}\,d-c}{u_{5}^{T}\,d-c}\,c_{t_{6}} \end{aligned}$$

According to the skyplot in (a), $c=\cos \,40^{\circ }$ and $u_{5}^{T}d=\cos \,60^{\circ }$ and $u_{6}^{T}d=\cos \,80^{\circ }$, which means that $\Vert c_{t_{5}}\Vert _{Q_{tt}}>\Vert c_{t_{6}}\Vert _{Q_{tt}}$, thus $\mathrm{P}_{\mathrm{CD}_{5}}>\mathrm{P}_{\mathrm{CD}_{6}}$. Since ${\bar{c}}_{5}\parallel {\bar{c}}_{6}$ and $\overline{{\mathcal {P}}}_{5}=\overline{{\mathcal {P}}}_{6}$, the difference between $\mathrm{P}_{\mathrm{CI}_{5}}$ and $\mathrm{P}_{\mathrm{CI}_{6}}$ for a given bias-to-noise ratio depends only on the difference between $\Vert c_{t_{5}}\Vert _{Q_{tt}}$ and $\Vert c_{t_{6}}\Vert _{Q_{tt}}$. Therefore, $\Vert c_{t_{5}}\Vert _{Q_{tt}}>\Vert c_{t_{6}}\Vert _{Q_{tt}}$ will also lead to $\mathrm{P}_{\mathrm{CI}_{5}}>\mathrm{P}_{\mathrm{CI}_{6}}$.

In (b), all the satellites except G4 locate *almost* on a cone with its axis shown as the *red* circle. If the satellites G1, G2, G3, G5 and G6 would have formed a *perfect* cone, then the contribution of the G4 observation to the misclosures would have been identically *zero*. This can be shown by proving that the fourth column of $B^{T}$, i.e., $c_{t_{4}}$, becomes a zero-vector. If the unit vectors $u_{i}$ for $i\ne 4$ lie on a cone with *d* being its symmetry axis, then for some scalar $c\in {\mathbb {R}}$ we have $u_{i}^{T}\,d=c$ (cf. 42). Multiplying the corresponding SPP design matrix *A* with $B^{T}$ from left and with $[d^{T},~c]^{T}$ from right, we arrive at

45 $$\begin{aligned} c_{t_{4}}\,(u_{4}^{T}\,d-c)=0 \end{aligned}$$

Since $u_{4}$ does not lie on the mentioned cone, then $u_{4}^{T}\,d\ne c$ implying that $c_{t_{4}}=0$, thus $\mathrm{P}_{\mathrm{CD}_{4}}=\mathrm{P}_{\mathrm{CI}_{4}}=0$. However, as the line-of-sights to the satellites G1, G2, G3, G5 and G6 do *not* form a perfect cone, i.e., $u_{i\ne 4}^{T}\,d\approx c$, the observation of satellite $G_{4}$ has a nonzero contribution to the misclosure vector resulting in nonzero values for $\mathrm{P}_{\mathrm{CD}_{4}}$ and $\mathrm{P}_{\mathrm{CI}_{4}}$. It can be seen that $\mathrm{P}_{\mathrm{CD}_{4}}$ and $\mathrm{P}_{\mathrm{CI}_{4}}$ are significantly smaller than, respectively, $\mathrm{P}_{\mathrm{CD}_{i\ne 4}}$ and $\mathrm{P}_{\mathrm{CI}_{i\ne 4}}$. To understand the distinct behavior of $\mathrm{P}_{\mathrm{CD}_{4}}$ compared to $\mathrm{P}_{\mathrm{CD}_{i\ne 4}}$, we look at $\Vert c_{t_{i}}\Vert _{Q_{tt}}$ given as

46 $$\begin{aligned} \Vert c_{t_{i}}\Vert _{Q_{tt}}\;=\;\sigma ^{-1}\;\left[ \dfrac{m}{m-1}+\Vert u_{i}-{\bar{u}}_{\ne i}\Vert ^{2}_{C_{xx\ne i}}\right] ^{-\frac{1}{2}} \end{aligned}$$

where $C_{xx\ne i}=\sum _{k\ne i}^{m}(u_{k}-{\bar{u}}_{\ne i})(u_{k}-{\bar{u}}_{\ne i})^{T}$ and ${\bar{u}}_{\ne i}=\frac{1}{m-1}\sum _{k\ne i}^{m}u_{k}$. The quadratic expression within the brackets can be worked out using the eigenvalue decomposition of $C_{xx\ne i}$ as

47 $$\begin{aligned} \Vert u_{i}-{\bar{u}}_{\ne i}\Vert ^{2}_{C_{xx\ne i}}=\sum _{j=1}^{3}\lambda ^{-1}_{j,i}\left[ (u_{i}-{\bar{u}}_{\ne i})^{T}\,v_{j,i}\right] ^{2} \end{aligned}$$

in which $\lambda _{j,i}$ and $v_{j,i}$ for $j=1,2,3$ are, respectively, the eigenvalues and the corresponding eigenvectors of $C_{xx\ne i}$. Assuming $\lambda _{1,i}\ge \lambda _{2,i}\ge \lambda _{3,i}$, for a given value of $\Vert u_{i}-{\bar{u}}_{\ne i}\Vert$, (47) achieves its maximum when $(u_{i}-{\bar{u}}_{\ne i})\parallel v_{3,i}$. In the following, we check $\lambda _{3,i}$ (the minimum eigenvalue), the angle between $(u_{i}-{\bar{u}}_{\ne i})$ and $v_{3,i}$ (eigenvector corresponding with the minimum eigenvalue), and $\Vert u_{i}-{\bar{u}}_{\ne i}\Vert$ for $i=1,\ldots ,6$.

- $\lambda _{3,i}$: For $i=4$, since $u_{j\ne 4}^{T}\,d\approx c$, it can be concluded that $v_{3,4}$ is almost parallel to *d* and $\lambda _{3,4}\approx 0$. This implies that $\lambda _{3,4}^{-1}$ is extremely large. For $i\ne 4$, among the five remaining satellites, still there are four unit vectors which satisfy $u_{j\ne i,4}^{T}\,d\approx c$. Therefore, the eigenvector $v_{3,i\ne 4}$ does not deviate too much from the direction *d*. However, due to the presence of satellite G4 not lying on the mentioned cone, $\lambda _{3,i\ne 4}$ is much larger than zero, implying that $\lambda _{3,i\ne 4}^{-1}$ is much smaller than $\lambda _{3,4}^{-1}$.
- The angle between $(u_{i}-{\bar{u}}_{\ne i})$ and $v_{3,i}$: As shown in the skyplot in (b), while $u_{4}$ is almost parallel to $v_{3,4}$, $u_{i\ne 4}$ makes an almost $56^{\circ }$ with $v_{3,i\ne 4}$ (almost parallel to *d*). For the geometry shown in (b), ${\bar{u}}_{\ne 4}$ is almost parallel to $v_{3,4}$, whereas this is not the case with ${\bar{u}}_{\ne i}$ ($i\ne 4$). Therefore, we have $(u_{4}-{\bar{u}}_{\ne 4})\parallel v_{3,4}$.
- $\Vert u_{i}-{\bar{u}}_{\ne i}\Vert$: We can write $\Vert u_{i}-{\bar{u}}_{\ne i}\Vert ^{2}=1+\Vert {\bar{u}}_{\ne i}\Vert ^{2}-2u_{i}^{T}{\bar{u}}_{\ne i}$. Since ${\bar{u}}_{\ne i}$ is computed based on five out of six unit direction vectors, its norm does not change too much for different *i*. Therefore, $\Vert u_{i}-{\bar{u}}_{\ne i}\Vert$ gets its minimum value for $i=4$ as $u_{4}$ is almost parallel to ${\bar{u}}_{\ne 4}$. However, $\Vert u_{4}-{\bar{u}}_{\ne 4}\Vert <\Vert u_{i}-{\bar{u}}_{\ne i}\Vert$ is overcompensated by $\lambda _{3,4}^{-1}>\lambda _{3,i}^{-1}$.

Given the above explanation, $\Vert u_{4}-{\bar{u}}_{\ne 4}\Vert ^{2}_{C_{xx\ne 4}}$ is much larger than $\Vert u_{i}-{\bar{u}}_{\ne i}\Vert ^{2}_{C_{xx\ne i}}$, and $\Vert c_{t_{4}}\Vert _{Q_{tt}}$ is thus much smaller compared to $\Vert c_{t_{i}}\Vert _{Q_{tt}}$. This explains that the CD probability of ${\mathcal {H}}_{4}$ is much smaller than that of ${\mathcal {H}}_{i\ne 4}$. As $\overline{{\mathcal {P}}}_{4}$ and the orientation of ${\bar{c}}_{4}$ within it are similar to those of ${\mathcal {H}}_{i}$ with $i=1,3,6$ and poorer than ${\mathcal {H}}_{i}$ with $i=2,5$, then $\Vert c_{t_{i\ne 4}}\Vert _{Q_{tt}}>\Vert c_{t_{4}}\Vert _{Q_{tt}}$ can also explain why $\mathrm{P}_{\mathrm{{CI}}_{i\ne 4}}>\mathrm{P}_{\mathrm{{CI}}_{4}}$.

## Conclusion and summary

In this contribution, we presented datasnooping in the context of the DIA method, discussed its decision probabilities for detection and identification and showed what options one has available when two or more of the alternative hypotheses are nonseparable.

In our discussion, we emphasized the central role that is played by the partitioning of misclosure space, both in the formation of the decision probabilities and in the construction of the DIA estimator. In case of datasnooping, the partitioning is determined by the row vectors of the basis matrix of the null space of $A^{T}$. Through this partitioning, the distribution of the misclosure vector can be used to determine the correct detection (CD) and correct identification (CI) probabilities of each of the alternative hypotheses. These probabilities can be ‘inverted’ to determine their corresponding minimal biases, the minimal detectable bias (MDB) and the minimal identifiable bias (MIB). We highlighted their difference by showing the difference between their corresponding contributing factors. In particular, it should be realized that the MDB provides information about correct detection and *not* about correct identification. A high probability of correct detection does namely not necessarily imply a high probability of correct identification, unless one is dealing with the special case of having only one single alternative hypothesis.

In the identification step, one has to ascertain whether or not all the hypotheses are identifiable. Identification of hypotheses becomes problematic if the misclosure vector has the *same* distribution under *different* hypotheses. We discussed the options one can choose from in terms of ‘remeasurement’, ‘adaptation’ or stating that the solution is ‘unavailable’. Of these, the adaptation step is the most involved. By means of an equivalence between the nonseparability of hypotheses and the inestimability of parameters (cf. Theorem 1), we demonstrated that one can forget about adapting ${\hat{x}}_{0}$ for hypotheses that are nonseparable. However, as this concerns the *complete* vector *x* and not necessarily functions of *x*, we also demonstrated that functions of *x* may exist for which adaptation is still possible (cf. Theorem 2). It was shown how this adaptation looks like and how it changes the structure of the DIA estimator.

We applied the theory to selected examples so as to illustrate and explain the performance of the various elements of DIA-datasnooping. Three different cases were discussed in detail: height-difference observations of a leveling network, distance measurements of a horizontal geodetic network and pseudorange measurements between a single ground station and GPS satellites. We analyzed and illustrated how geometry changes in the measurement setup affect the testing procedure, including its partitioning of the misclosure space, and the corresponding CD probabilities (MDB) and CI probabilities (MIB). We also demonstrated that for a given bias-to-noise ratio and a false alarm probability, the ordering of the CD probabilities of the alternative hypotheses is not necessarily the same as that of their CI probabilities. And we showed, if two alternative hypotheses, say ${\mathcal {H}}_{i}$ and ${\mathcal {H}}_{j}$, are not distinguishable, that the testing procedure may have different levels of sensitivity to ${\mathcal {H}}_{i}$-biases compared to the same ${\mathcal {H}}_{j}$-biases.

## Appendix

### Proof of Lemma 1

That $\cup _{i=1}^{k}{\mathcal {P}}_{i}={\mathbb {R}}^{r}/{\mathcal {P}}_{0}$ follows from the fact that since any $t\in {\mathbb {R}}^{r}/{\mathcal {P}}_{0}$ produces a vector of *k* test statistics $w_{i}$ ($i=1,\ldots ,k$), for any such *t* there is a region ${\mathcal {P}}_{i}$ in which it lies for some *i*. ‘if’ part: If $c_{t_{i}}\nparallel c_{t_{j}}$ for any $i\ne j$, then only one of the test statistics $w_{i}$ ($i=1,\ldots ,k$) will produce the maximum absolute value. To prove this, let $|w_{i}|=|w_{j}|=\underset{k=\{1,\ldots ,m\}}{\max }{|w_{k}|}$ for $i\ne j$ and for some $t\in {\mathbb {R}}^{r}/{\mathcal {P}}_{0}$. Then

48 $$\begin{aligned} (\Vert c_{t_{i}}\Vert ^{-1}_{Q_{tt}}c_{t_{i}}-\Vert c_{t_{j}}\Vert ^{-1}_{Q_{tt}}c_{t_{j}})^{T}\,Q_{tt}^{-1}\,t\;=\;0 \end{aligned}$$

from which follows that $t=0$ cannot be a solution since $t\in {\mathbb {R}}^{r}/{\mathcal {P}}_{0}$, nor can *t* be orthogonal to $Q_{tt}^{-1}(\Vert c_{t_{i}}\Vert ^{-1}_{Q_{tt}}c_{t_{i}}-\Vert c_{t_{j}}\Vert ^{-1}_{Q_{tt}}c_{t_{j}})$ since the probability of that occurrence is zero, thus leaving as only possibility that $c_{t_{i}}\parallel c_{t_{j}}$, which contradicts our earlier assumption of $c_{t_{i}}\nparallel c_{t_{j}}$. Therefore, any $t\in {\mathbb {R}}^{r}/{\mathcal {P}}_{0}$ lies in only one of the regions ${\mathcal {P}}_{i}$ ($i=1,\ldots ,k$), revealing that $\cup _{i=1}^{k}{\mathcal {P}}_{i}={\mathbb {R}}^{r}/{\mathcal {P}}_{0}$ and ${\mathcal {P}}_{i}\cap {\mathcal {P}}_{j}=\emptyset$ for any $i\ne j$. As a result, the $m+1$ regions ${\mathcal {P}}_{i}$ form a partitioning of ${\mathbb {R}}^{r}$.

For the ‘only if’ part we have: Let us assume that $c_{t_{i}}\parallel c_{t_{j}}$ for $i\ne j$. Then, given (7), we have $|w_{i}|=|w_{j}|$ for any $t\in {\mathbb {R}}^{r}$. This reveals that ${\mathcal {P}}_{i}={\mathcal {P}}_{j}$ which contradicts our earlier assumption of ${\mathcal {P}}_{i}\cap {\mathcal {P}}_{j}=\emptyset$. Therefore, $c_{t_{i}}\nparallel c_{t_{j}}$ for any $i\ne j$. $\square$

### Proof of Lemma 2

Consider the 2-vector ${\bar{t}}$ with a length of ${\bar{l}}$ which makes an angle of ${\bar{\beta }}_{i}$ with ${\bar{c}}_{i}$ measured counterclockwise. Therefore, we have

49 $$\begin{aligned} \begin{array}{lll} {\bar{t}}&{}=&{}{\bar{l}}\;\left[ \begin{array}{lr}\cos {\bar{\beta }}_{i} &{} -\sin {\bar{\beta }}_{i}\\[2mm] \sin {\bar{\beta }}_{i}&{} \cos {\bar{\beta }}_{i} \end{array}\right] \,{\bar{c}}_{i} \end{array} \end{aligned}$$

Given the above equation and the orientation of ${\bar{c}}_{i}$ w.r.t. the straight borders of $\overline{{\mathcal {P}}}_{i}$ in (11), one can write

50

with

51

With (49), one can also obtain the joint PDF of $[{\bar{l}},{\bar{\beta }}_{i}]^{T}$ from $f_{{\bar{t}}}({\tau }|{\mathcal {H}}_{i})$ through the PDF transformation rule as

52 $$\begin{aligned} f_{{\bar{l}},{\bar{\beta }}_{i}}(l,\beta |{\mathcal {H}}_{i})= & {} \,l\,f_{{\bar{t}}}({\tau }(l,\beta )|{\mathcal {H}}_{i})\nonumber \\= & {} \,\dfrac{l}{2\pi }\exp \left\{ -\,\dfrac{1}{2}\,(l^{2}+\Vert \mu _{{\bar{t}}_{i}}\Vert ^{2}-2l\Vert \mu _{{\bar{t}}_{i}}\Vert \cos \beta )\right\} \nonumber \\ \end{aligned}$$

with $\Vert \mu _{{\bar{t}}_{i}}\Vert =|b_{i}|\,\Vert c_{t_{i}}\Vert _{Q_{tt}}$. Equations (50) and (52) enable us to express the CI probability in terms of ${\bar{l}}$ and ${\bar{\beta }}_{i}$ as

53 $$\begin{aligned} \mathrm{P}_{\mathrm{CI}_{i}}= & {} \int _{\overline{{\mathcal {P}}}_{i}}\;f_{{\bar{t}}}({\tau }|{\mathcal {H}}_{i})\;\mathrm{d}\tau \nonumber \\= & {} \int _{{\mathcal {L}}_{i}}\,\int _{\sqrt{k_{\alpha }}}^{\infty }\;f_{{\bar{l}},{\bar{\beta }}_{i}}(l,\beta )\;\mathrm{d}l\mathrm{d}\beta \end{aligned}$$

Substitution of (52) into (53), and then taking the derivative w.r.t. $\beta _{i,1}$, we achieve

54 $$\begin{aligned}&\displaystyle {\int }_{\sqrt{k_{\alpha }}}^{\infty }\,\dfrac{l}{2\pi }\exp \left\{ -\dfrac{1}{2}\,(l^{2}+\Vert \mu _{\bar{t}}\Vert ^{2})\right\} \nonumber \\&\quad \times \,[\exp \left\{ l\Vert \mu _{\bar{t}}\Vert \cos \beta _{i,1}\right\} -\exp \left\{ l\Vert \mu _{\bar{t}}\Vert \cos (\beta _{i}-\beta _{i,1})\right\} \nonumber \\&\quad +\,\exp \left\{ -l\Vert \mu _{\bar{t}}\Vert \cos \beta _{i,1}\right\} -\exp \left\{ -l\Vert \mu _{\bar{t}}\Vert \cos (\beta _{i}-\beta _{i,1})\right\} ]\; \mathrm{d}l \end{aligned}$$

Setting the above derivative equal to zero, a set of solutions for $\beta _{i,1}$ is given by

55 $$\begin{aligned} \beta _{i,1}=k\pi +\frac{1}{2}\beta _{i},\qquad k\in {\mathbb {Z}} \end{aligned}$$

Since $\beta _{i,1}<\pi$, then the only valid solution is $\beta _{i,1}=\frac{1}{2}\beta _{i}$. To check whether this critical point is the maximizer of (53), we compute the derivative of the expression in (54) at $\beta _{i,1}=\frac{1}{2}\beta _{i}$, which is

56 $$\begin{aligned}&\displaystyle {\int }_{\sqrt{k_{\alpha }}}^{\infty }\,\dfrac{l^{2}\Vert \mu _{{\bar{t}}}\Vert }{2\pi }\exp \left\{ -\dfrac{1}{2}\,(l^{2}+\Vert \mu _{{\bar{t}}}\Vert ^{2})\right\} \nonumber \\&\quad \times \,\left[ -2\sin \frac{\beta _{i}}{2}\exp \left\{ l\Vert \mu _{{\bar{t}}}\Vert \cos \frac{\beta _{i}}{2}\right\} \right. \nonumber \\&\quad \left. +2\sin \frac{\beta _{i}}{2}\exp \left\{ -l\Vert \mu _{{\bar{t}}}\Vert \cos \frac{\beta _{i}}{2}\right\} \right] \;\mathrm{d}l \end{aligned}$$

Since $0<\beta _{i}<\pi$, then $\sin \frac{\beta _{i}}{2}>0$ and $\cos \frac{\beta _{i}}{2}>0$. These, in tandem with $l\Vert \mu _{{\bar{t}}}\Vert >0$ and the fact that $\exp \{\cdot \}$ is a ‘positive’ increasing function, imply that the expression in (56) is ‘negative.’ Thus, $\beta _{i,1}=\frac{1}{2}\beta _{i}$ is the maximizer of (53). $\square$

### Proof of Theorem 1

We start with the ‘if’ part: If there exists a nonzero matrix $X\in {\mathbb {R}}^{n\times q}$ such that $A\,X=C_{i}-\,C_{j}\,X_{i,j}$ for some invertible matrix $X_{i,j}\in {\mathbb {R}}^{q\times q}$, then multiplying both sides of the equation with $B^{T}$ from left gives

57 $$\begin{aligned} B^{T}\,C_{i}\;=\;B^{T}\,C_{j}\,X_{i,j} \end{aligned}$$

For the ‘only if’ part we have: If $B^{T}\,C_{i}\;=\;B^{T}\,C_{j}\,X_{i,j}$ for some invertible matrix $X_{i,j}\in {\mathbb {R}}^{q\times q}$, then two conclusions can be made: 1. $C_{i}=C_{j}\,X_{i,j}$ which is not possible as it contradicts our assumption that rank $([C_{i}~~C_{j}])>q$; 2. $(C_{i}-\,C_{j}\,X_{i,j})\in {\mathcal {R}}(A)$. Therefore, there exists a nonzero matrix $X\in {\mathbb {R}}^{n\times q}$ such that $A\,X=C_{i}-\,C_{j}\,X_{i,j}$ or equivalently

58 $$\begin{aligned}{}[A~~C_{i}~~C_{j}]\,\left[ \begin{array}{c}XI_{q}\\ X_{i,j}\end{array}\right] \;=\;0 \end{aligned}$$

$$\square $$

### Proof of Theorem 2

Since $\theta$ is a linear function of *x* and not *b*, we reduce the observational model in (24) to one containing only the unknown parameters *x* as

59 $$\begin{aligned} E({C}^{\perp ^{T}}y)\;=\;{C}^{\perp ^{T}}A\,x;\quad D(y)\;=\;{C}^{\perp ^{T}}Q_{yy}{C}^{\perp } \end{aligned}$$

where ${C}^{\perp }$ is a basis matrix of the orthogonal complement of the range space of *C*. Let *V* be a basis matrix of the null space of ${C}^{\perp ^{T}}A$. Furthermore, let *S* be a full-rank matrix of which the range space is complementary to that of *V*. Therefore, $[S~~V]\in {\mathbb {R}}^{n\times n}$ is an invertible matrix, thus a basis matrix of ${\mathbb {R}}^{n}$. This indicates that any vector $x\in {\mathbb {R}}^{n}$ can be parametrized as

60 $$\begin{aligned} x\;=\;S\,x_{S} \;+\;V\,x_{V} \end{aligned}$$

(i) We now show that $\theta$ is unbiased estimable under (59) iff $F^{T}V=0$. ‘if’ part: If $F^{T}V=0$, then $\theta =F^{T}S\,x_{S}$. Substituting (60) into (59), as ${C}^{\perp ^{T}}A\,V=0$, gives us

61 $$\begin{aligned} E({C}^{\perp ^{T}}y)\;=\;{C}^{\perp ^{T}}A\,S\,x_{S};\quad D(y)\;=\;{C}^{\perp ^{T}}Q_{yy}{C}^{\perp } \end{aligned}$$

which shows that $x_{S}$ as well as any linear function of it like $\theta$ are unbiased estimable under (59). ‘only if’ part: Equation (61) shows that $x_{V}$ is *not* estimable. Therefore, for $\theta =F^{T}x$ to be estimable in (59), it should be a function of only $x_{S}$ and not $x_{V}$, i.e., $F^{T}V=0$ and $\theta =F^{T}S\,x_{S}$.

(ii) The BLUE of $x_{S}$ based on (61) is as follows

62 $$\begin{aligned} {\hat{x}}_{S}\;=\;(S^{T}{\bar{A}}^{T}Q_{yy}^{-1}{\bar{A}}S)^{-1}S^{T}{\bar{A}}^{T}Q_{yy}^{-1}y \end{aligned}$$

in which ${\bar{A}}=P^{\perp }_{C}A$ and $P^{\perp }_{C}=I_{m}-C(C^{T}Q_{yy}^{-1}C)^{-1}C^{T}Q_{yy}^{-1}$. It can be shown (Teunissen and Khodabandeh 2013) that the BLUE of $x_{S}$ based on (1), ${\hat{x}}_{S_{0}}$, is linked to ${\hat{x}}_{S}$ as

63 $$\begin{aligned} {\hat{x}}_{S_{0}}\;=\;{\hat{x}}_{S}-Q_{{\hat{x}}_{S},t}Q_{tt}^{-1}t \end{aligned}$$

where *t* and $Q_{tt}$ are given in (4) and $Q_{{\hat{x}}_{S},t}$ is the covariance between ${\hat{x}}_{S}$ and *t* which can be computed given (4) and (62). Multiplying both sides of the above equation with $F^{T}S$ from left gives the link between ${\hat{\theta }}=F^{T}S\,{\hat{x}}_{S}$ and ${\hat{\theta }}_{0}=F^{T}S\,{\hat{x}}_{S_{0}}$. The link between their variances is also obtained using the error propagation law. $\square$

***Numerical evaluation of***${\varvec{P}}_{{{\varvec{CI}}}_{{\varvec{i}}}}$***in*** (18): In this study, given Eqs. (5)–(8), the CI probability under ${\mathcal {H}}_{i}$ (cf. 18) is computed through the following steps.

- Generate *n* samples of *t* from the normal distribution ${\mathcal {N}}(\mu _{t_{i}}=b_{i}c_{t_{i}},Q_{tt})$.
- Compute $\Vert t\Vert ^{2}_{Q_{tt}}$ for all the samples. Single out those samples satisfying $\Vert t\Vert ^{2}_{Q_{tt}}>k_{\alpha }$ (i.e., $t\notin {\mathcal {P}}_{0}$) and collect them in a set denoted by $\Omega _{\mathrm{CD}}$.
- For each sample in $\Omega _{\mathrm{CD}}$, compute the w-tests $w_{j}$ for $j=1,\ldots ,k$ in (7). Count the number of samples for which $|w_{i}|\ge |w_{j}|$ for any $j\ne i$ (i.e., $t\in {\mathcal {P}}_{i}$), and denote it by $n_{\mathrm{CI}}$.
- Compute the CI probability under ${\mathcal {H}}_{i}$ as $P_{\mathrm{CI}_{i}}=\frac{n_{\mathrm{CI}}}{n}$. $\square$
